# Supplementary material for: Exploring ethnic minority women’s experiences of maternity care during the SARS-CoV-2 pandemic: a qualitative study
Source: BMJ Open. 2021 Sep 6;11(9):e050666. doi: 10.1136/bmjopen-2021-050666 (PMC8423508; doi:10.1136/bmjopen-2021-050666)
Supplement: Author's manuscript [file bmjopen-2021-050666.draft_revisions.pdf]

BMJ Open

BMJ Open is committed to open peer review. As part of this commitment we make the peer review history of every article we publish publicly available.

When an article is published we post the peer reviewers' comments and the authors' responses online. We also post the versions of the paper that were used during peer review. These are the versions that the peer review comments apply to.

The versions of the paper that follow are the versions that were submitted during the peer review process. They are not the versions of record or the final published versions. They should not be cited or distributed as the published version of this manuscript.

BMJ Open is an open access journal and the full, final, typeset and author-corrected version of record of the manuscript is available on our site with no access controls, subscription charges or pay-per-view fees (http://bmjopen.bmj.com).

If you have any questions on BMJ Open's open peer review process please email info.bmjopen@bmj.com

BMJ Open

Exploring ethnic minority women's experiences of maternity care during the SARS-CoV-2 pandemic: a qualitative study

Journal:	*BMJ Open*
Manuscript ID	bmjopen-2021-050666
Article Type:	Original research
Date Submitted by the Author:	27-Feb-2021
Complete List of Authors:	John, Jeeva; The University of Edinburgh Usher Institute of Population Health Sciences and Informatics,<br>Curry, Gwenetta; The University of Edinburgh Usher Institute of Population Health Sciences and Informatics<br>Cunningham-Burley, Sarah; The University of Edinburgh Usher Institute of Population Health Sciences and Informatics, Public Health Sciences
Keywords:	QUALITATIVE RESEARCH, COVID-19, HEALTH SERVICES ADMINISTRATION & MANAGEMENT, Organisation of health services < HEALTH SERVICES ADMINISTRATION & MANAGEMENT, Quality in health care < HEALTH SERVICES ADMINISTRATION & MANAGEMENT, OBSTETRICS

SCHOLARONE™
Manuscripts

I, the Submitting Author has the right to grant and does grant on behalf of all authors of the Work (as defined in the below author licence), an exclusive licence and/or a non-exclusive licence for contributions from authors who are: i) UK Crown employees; ii) where BMJ has agreed a CC-BY licence shall apply, and/or iii) in accordance with the terms applicable for US Federal Government officers or employees acting as part of their official duties; on a worldwide, perpetual, irrevocable, royalty-free basis to BMJ Publishing Group Ltd ("BMJ") its licensees and where the relevant Journal is co-owned by BMJ to the co-owners of the Journal, to publish the Work in this journal and any other BMJ products and to exploit all rights, as set out in our licence.

The Submitting Author accepts and understands that any supply made under these terms is made by BMJ to the Submitting Author unless you are acting as an employee on behalf of your employer or a postgraduate student of an affiliated institution which is paying any applicable article publishing charge ("APC") for Open Access articles. Where the Submitting Author wishes to make the Work available on an Open Access basis (and intends to pay the relevant APC), the terms of reuse of such Open Access shall be governed by a Creative Commons licence – details of these licences and which Creative Commons licence will apply to this Work are set out in our licence referred to above.

Other than as permitted in any relevant BMJ Author's Self Archiving Policies, I confirm this Work has not been accepted for publication elsewhere, is not being considered for publication elsewhere and does not duplicate material already published. I confirm all authors consent to publication of this Work and authorise the granting of this licence.

For peer review only

Exploring ethnic minority women's experiences of maternity care during the SARS-CoV-2 pandemic: a qualitative study

Dr. Jeeva John MBChB[1] (Corresponding author: jeeva.john@ed.ac.uk, Ph: 07792562199) Dr. Gwenetta Curry[2], Prof. Sarah Cunningham-Burley[1]

1- The University of Edinburgh Usher Institute of Population Health Sciences and Informatics, Centre for Biomedicine, Self and Society, Edinburgh, Edinburgh, UK

2- The University of Edinburgh Usher Institute of Population Health Sciences and Informatics, Usher Institute, Edinburgh, Edinburgh, UK

Running title: Ethnic minority maternity care during Covid-19

For peer review only

Abstract

Objective:

To explore ethnic minority women's experiences of maternity care during the SARS-CoV-2 pandemic.

Design:

Qualitative study.

Population:

Sixteen Black, Asian and Minority Ethnic women who were pregnant, or had delivered within 6 weeks prior to interview in a predominantly urban Scottish health board area.

Methods:

Thematic analysis of semi-structured in-depth interviews.

Results

Four themes were identified: 'communication', 'interactions with health care professionals', 'racism' and 'the pandemic effect'. Each theme had relevant sub-themes. 'Communication' encompassed respect, accent bias, language barrier and cultural dissonance; 'interactions with health care professionals': continuity of care, empathy, informed decision making and dissonance with other health care systems; 'racism' was deemed to be institutional, interpersonal and internalised; and 'the pandemic effect' consisted of isolation, psychological impact and barriers to access of care.

Main outcome measures:

To explore the experiences of pregnancy, childbirth, antenatal and postnatal care in women belonging to ethnic minorities and to identify any specific challenges that these women faced during the SARS-CoV-2 pandemic.

Conclusions

This study provides insight into the specific challenges faced by ethnic minority women in pregnancy, which intersect with the unique problems posed by the ongoing SARS-CoV-2 pandemic to potentially widen existing ethnic disparities in maternal outcomes and experiences of maternity care.

Funding:

Ethical approval for this study was granted by the Research Ethics Committee of West of Scotland (20/WS/0168) on 26/11/2020.

For peer review only

Strengths and limitations of this study

- This study addresses a gap in the literature by using qualitative methods to provide an in-depth exploration of minority ethnic women's experiences of maternity care during the SARS-CoV-2 pandemic.

- This study explores the perspectives of pregnant ethnic minority women at different stages of pregnancy, as well as the postnatal period.

- A flexible topic guide was developed using existing literature, in collaboration with an obstetrician, a social scientist and a race and ethnicity lecturer, bringing together different ideologies to a complex issue.

- The interviewer is an obstetrician, and although none of the participants were known to the interviewer clinically, they were aware of the interviewer's role, and it is important to acknowledge that this could have had an indirect effect on participant responses.

For peer review only

Introduction

Whilst the overall Maternal Mortality Rate (MMR) in the U.K. has remained relatively low over the past decade[1], the gulf in maternal outcomes between White women and women from ethnic minority backgrounds continues to expand. The latest national confidential enquiry into maternal deaths (MBRRACE-U.K) report showed significant racial variations in maternal mortality. Black women were four times, while Asian women were two times as likely to die as White women during pregnancy, delivery or postnatally between 2016 and 2018.[2]

In previous country wide surveys of maternity care, ethnic minority women were more likely to report poor patient experience; in some cases attributed to stereotyping and racism.[3]

The SARS-CoV-2 pandemic has not only shone a spotlight on these disparities but may have exacerbated them.[4] A key component in establishing equality of maternal healthcare provision, is the examination of women's experiences of these services. There is little qualitative research on the lived experiences of ethnic minority women during or immediately following pregnancy in the U.K. This study aimed to explore the experiences of pregnancy, childbirth, antenatal and postnatal care in women belonging to ethnic minorities and to identify any specific challenges that these women faced during the SARS-CoV-2 pandemic.

For peer review only

Methods

[illegible]

Participant Characteristics and Recruitment

This study was performed in all of the community midwifery hubs and obstetric units within a predominantly urban Scottish health board. Purposive sampling was implemented to recruit women who self-identified as ethnic minority. Units were provided with study recruitment packs containing a participant information sheet, consent form and pre-paid envelope. Midwives were encouraged to share study information with ethnic minority patients and to provide them with recruitment packs. With their consent, participant details were securely passed onto researcher, JJ, who telephoned each patient 48 hours after receiving a recruitment pack. Participants who could not speak English were provided appropriate translations of study documents and interpreters were made available for interviews. JJ started telephone interviews by reconfirming participants' consent. Following the interview, participants were asked to post completed consent forms back to the researchers using prepaid envelopes. The interview topic guide was developed by all three authors, and was used to aid semi-structured interviews carried out by JJ.

Data Collection

Data collection occurred between December 2020 and January 2021. Audio-recorded interviews were transcribed verbatim by First *Class* Secretarial Services. Collected data was kept confidential by allocating a distinct code to each woman in order to protect anonymity. Data was collected until no new themes were identified and inductive thematic saturation was achieved.

Data Analysis

This study adopted a data analysis methodology based on thematic analysis.[5] Individual transcripts were read and re-read to enable researcher familiarity with the data collected, and to identify initial codes. Further analysis facilitated the categorisation of codes through grouping of initial codes and development of collective themes. Findings were collated into resulting themes, and illustrative quotes were highlighted. Data analysis was undertaken by JJ and GC acted as an external validator of the analysis process. A consensus discussion regarding theme development was undertaken by all three authors.

Patient and public involvement

There was no formal PPI group that assisted with this study. However, in the course of recruitment and interviews, advice from participants and health care professionals was sought and used to create a flexible interview topic guide. A summary of study findings will be available on the Centre for Biomedicine, Self and Society for general access, and study findings will also be disseminated amongst study participants with consent having been obtained at time of interview.

Details of ethics approval

Ethical approval for this study was granted by the Research Ethics Committee of West of Scotland (20/WS/0168).

Results

Sixteen women participated in telephone interviews (see Table 1 for sample characteristics).

Four principal themes were identified from interview data. These included communication, interactions with healthcare professionals, racism, and the pandemic effect. Each theme had subthemes, as presented in Figure 1.

Direct quotes relating to each theme are presented in Tables 2, 3, 4 and 5.

For peer review only

Communication

Respect

Participants who were satisfied with the maternal health system made reference to respectful communication. This was particularly important during episodes of high maternal stress (Table 2: *Respect,* P15). Participants concurred that community midwives and health visitors were considerate in their routine communication (Table 2: *Respect,* P1). A significant majority of the group conversely felt that they received disrespectful care during unsolicited hospital visits (Table 2: *Respect,* P11; P16).

Accent bias

All participants except one were first-generation immigrants with median duration of stay in the U.K. of nine years. The majority of the cohort had non "British" accents and identified bias due to accent as being a significant concern, sometimes perceived to impede access to emergency care, and prevent equality of maternal care received. One participant highlighted having a non "Westernised" accent as being interpreted as a proxy for lower socio-economic status and educational attainment, and that this was a unique barrier with regards to telephone consultations (Table 2: *Accent bias*, P12).

Language barrier

In this study, most participants spoke English fluently; only one required an interpreter. Despite the high standard of English spoken, most participants felt that language barriers were the most common cause of miscommunication between themselves and healthcare professionals. They concurrently felt they themselves were more likely to make inappropriate decisions regarding their healthcare as a result of misinterpretation (Table 2: *Language barrier*, P8). Healthcare professional misunderstanding due to language barriers was also described as a prime feature in barriers to effective communication (Table 2: *Language barrier*, P2; P9). In contrast, the participant who required an Arabic interpreter had not experienced any instances of miscommunication when an interpreter was present during pre-organised appointments. However, she admitted facing major communication challenges during unscheduled calls when an interpreter was not available. She identified a gap in recognition from healthcare professionals regarding this particular scenario, and reported that she often resorts to searching the internet for pregnancy advice even during emergencies due to anxiety surrounding communication (Table 2: *Language barrier*, P14).

Cultural dissonance

Cultural dissonance was identified as another significant barrier to effective communication. Cultural dissonance between participant and healthcare professional (Table 2: *Cultural dissonance*, P2) as well as between participants and their wider community (Table 2: *Cultural* dissonance, P8) both impacted

For peer review only

quality of communication equally. This could be in the form of religious discordance in routine clinical practice (Table 2: *Cultural* dissonance, P11) or misunderstanding of wider cultural context (Table 2: *Cultural dissonance*, P5).

Interactions with healthcare professionals

Quality of interaction with healthcare professionals was generally associated with four key domains: continuity of care; empathy; informed decision making; and dissonance with previous experiences of maternity care.

Continuity of care

Overall, participants felt they received good continuity of care throughout their pregnancy. Primiparous women particularly valued routine postnatal check-ups (Table 3: *Continuity of care*, P2). A common sentiment that arose in women requiring regular input from secondary care during the antenatal period was ineffective communication between their community midwives and hospital midwives or obstetricians, and vice-versa, sometimes resulting in omission of crucial clinical information (Table 3: *Continuity of care*, P7). In the women who experienced prolonged hospital admissions, the inability to discuss ongoing care with the same healthcare professionals led to varying information being given to the patient leading to dissolution of trust (Table 3: *Continuity of care*, P3).

Empathy

The most important aspect of interactions with healthcare professionals was the presence of empathy. In instances where the healthcare professional was deemed indifferent, participants often cited being hurried, feeling unheard, and uncared for, which negatively impacted the interaction, and the overall perception of maternity care (Table 3: *Empathy*, P3). Participants who experienced apathy in previous pregnancies recognised that the possibility of recurrence was a source of anxiety throughout subsequent pregnancies (Table 3: *Empathy*, P9; P12).

Informed decision making

Although all participants reported some level of information provision from healthcare providers regarding clinical decision making, almost everyone agreed that they would benefit from more thorough discussions. Most participants received information about their pregnancy in the form of signposting to books or websites but they expressed that their individual information needs would have been better met by one-to-one discussions (Table 3: *Informed decision making*: P6). Women who felt that their questions remained unanswered did not feel involved in shared decision making (Table 3: *Informed decision making:* P3).

For peer review only

Dissonance with other healthcare systems

Half of the study population had experienced maternity care out with the study health board, with a significant proportion having delivered outside the U.K. All participants who previously delivered outside the U.K. had experienced privatised healthcare. Women who previously delivered in other parts of the U.K. reported higher satisfaction with the study health board in the current pregnancy. Access to early pregnancy care, and shared decision making were some of the reasons provided for this. The majority of the participants who had delivered abroad felt that their previous pregnancy experiences were generally better compared to care received in this health board. Reasons given for this discordance varied amongst individual participants, and included; improved access to ultrasound scans, availability of medical specialists, and treatment by healthcare providers (Table 3: *Dissonance with other healthcare systems*, P12; P14). A high proportion of primiparous women had sought advice from relatives, friends and/or healthcare professionals in their country of birth (Table 3: *Dissonance with other healthcare systems*, P3).

Racism

Institutional

Institutional racism was highlighted as a significant issue in pregnancy care by most of the participants. A small proportion of Black participants concomitantly worked in healthcare, and were able to provide examples of racial discrimination against themselves from their colleagues (Table 4: *Institutional*, P10). Some of the participants expressed distrust in maternal healthcare due to their concerns that medical research and treatments are tailored for their White counterparts (Table 4: *Institutional*, P8). Participants who had experienced institutional racism were also likely to perceive barriers to accessing healthcare compared to White women (Table 4: *Institutional*, P12).

Interpersonal

Most women gave examples of acts of racism against their friends, or family rather than themselves (Table 4: *Interpersonal*, P6; P12).

Internalised

In participants who expressed internalised racist beliefs, these almost always affected health behaviours. In one example, a significant delay in diagnosis of a common condition led one participant to believe that the main problem was her lack of assertiveness compared to White women (Table 4: *Internalised*, P6). In another case, a participant felt that she did not have the right to question the healthcare she was receiving, being originally from a low-income country (Table 4: *Internalised,* P11). Yet another participant strongly believed that disparities in maternal health outcomes could be explained by physiological differences between ethnic groups (Table 4: *Internalised*, P7).

For peer review only

The pandemic effect

Isolation

Most women reported feeling isolated during their pregnancy due to features specific to the SARS-CoV-2 pandemic. This was particularly a problem for those who felt that they would have benefitted from the presence of a companion when important information relating to their pregnancy was being relayed to them (Table 5: *Isolation*, P5). Although most women were understanding of the limitations posed by the pandemic, a significant proportion expressed loneliness exacerbated by not being able to engage with their usual pregnancy support networks, and did not feel that virtual groups mitigated this effect (Table 5: *Isolation,* P2; P3).

Psychological impact

The detrimental effect of the pandemic on mental health during pregnancy was highlighted by all participants. Women expressed feeling more anxious, fearful, and lacking autonomy in comparison to their previous pregnancies. This sentiment was particularly emphasized by the participants who had little or no family members nearby (Table 5: *Psychological impact*, P11; P13). The majority of participants reported that they were not routinely asked about their mental health in relation to the pandemic by healthcare professionals. Fewer still were signposted to appropriate support groups for help.

Barriers to access of care

Most participants had some of their routine face-to-face appointments replaced by telephone calls. The majority felt that virtual appointments were not as effective, especially during unscheduled care (Table 5: *Barriers to access of care*, P5). Women expressed uncertainty surrounding the accuracy of information relayed via telephone. (Table 5: *Barriers to access of care*, P6). In circumstances where participants did seek physical consultations, they experienced barriers, and often had to repeatedly call in order to be seen (Table 5: *Barriers to access of care*, P10; P11). On the contrary, some women delayed seeking medical help due to apprehensions surrounding contracting SARS-CoV-2 (Table 5: *Barriers to access of care*, P14).

Discussion

Main findings

This study focussed on the lived experiences during the SARS-CoV-2 pandemic of minority ethnic women who were pregnant, or had delivered within 6 weeks prior to interview. There were four emergent themes including communication, interactions with healthcare professionals, racism, and the effect of the pandemic, with further sub-themes identified. Although many of the issues identified are

For peer review only

not unique to minority ethnic women, the findings emphasise previous study results.[6,7,8] The systemic inadequacies highlighted in maternity care provision for women from ethnic minority backgrounds have been exacerbated by the SARS-CoV-2 pandemic.

Strengths and limitations

This is the first qualitative study, to our knowledge, which explores the maternity experiences of women from ethnic minority backgrounds during the SARS-CoV-2 pandemic in the U.K. The interviewer, JJ, is an obstetrician, and although none of the participants were known to JJ clinically, they were aware of the interviewer's role, and it is important to acknowledge that this could have had an indirect effect on participant responses.

Interpretations

The proportion of births to migrant women has increased annually in line with growing immigration, from 11.6 percent in 1990[9] to 28.2 percent in 2019.[10] Adequate and appropriate healthcare provision for ethnic minority groups has long been recognised as integral to safe maternity care.[11] The Equality Act 2010 states that NHS treatment and care, including maternal healthcare, should be equitable and no person should be discriminated against on the basis of their ethnicity.[12] Despite recognition of issues specific to this cohort, more than a decade later, ethnic minority women continue to suffer from worse pregnancy outcomes. The Right to Health document decrees that the four key elements of universal right to healthcare are that governments must ensure that healthcare services are ethically and culturally acceptable to all, accessible to all, available in sufficient quantity, and of good quality.[13] This study provides evidence to support that more needs to be done to guarantee that all ethnic minority women receive culturally acceptable, accessible, and equitable maternal healthcare in the U.K not only to tackle existing disparities but also to combat the additional detrimental effects of the SARS-CoV-2 pandemic.

The issues raised are not exclusive to ethnic minority women however, it is plausible that the need for support, effective communication, and good quality care are not met to a greater degree in this cohort, and that these long standing issues are exacerbated to a larger extent amongst these women as a result of the SARS-CoV-2 pandemic.

Good communication forms the foundation of good clinical care, and therefore, it is unsurprising that issues surrounding different aspects of communication were identified as a key theme. It is striking that although the majority of this group were fluent in English, they still identified it as a contributing problem, which mirrors previous studies' conclusions that language proficiency does not always facilitate a good pregnancy experience.[14,15]These issues are likely to be amplified with the changes in

maternal care provision during the SARS-CoV-2 pandemic, such as the predominance of telephone consultations, increasing risk of misunderstanding and misinformation. Communication, or lack thereof, played a major role in participants' perceptions of whether they were receiving acceptable care. This consisted of routine or emergency interactions with midwives, obstetricians, general practitioners and health visitors. The majority of participants reported that regular communication with their community midwives and health visitors was excellent, however collective areas of improvement were suggested for dealings with secondary care staff during emergency visits.

Despite overlap, interactions with healthcare professionals is treated as a separate theme to communication to highlight the role of the healthcare professional in providing maternal care beyond delivery of information. A unique sub-theme that arose in this study is the effect that previous maternal care outside the U.K. has on the perception of maternal health services during the current pregnancy. Women who had not delivered elsewhere were still likely to discuss their care with friends, family, or healthcare professionals from their country of birth where practices differed. The discordance in maternal healthcare proved a source of worry, and remained unaddressed by their healthcare providers. A broader understanding of variations in maternal care is vital to provide reassurance. Although not specific to ethnic minority women, the problems of continuity of care between primary and secondary care identified in this study, are likely to be aggravated by the changes posed by the SARS-CoV-2 pandemic.

As a general trend, duration of time spent in the U.K. was associated with awareness of racism impacting maternal health outcomes, and personal experience of racial discrimination. Sadly, the majority of participants were able to narrate examples of their friends, family members or wider community who had experienced racial discrimination both within and out with the context of healthcare in the U.K. Most women reported personal health behaviours that they had developed in response to others' experiences of discrimination. Previous studies have implicated patients' own experiences of racism in poor maternal health[16,17] however, the influence of health behaviour modification as a consequence of cognisance of discrimination that others face, as highlighted by this study, is not well explored.

All women recruited to this study had experienced a significant portion of their maternity care during lockdown restrictions due to the SARS-CoV-2 pandemic. Only two women in this study were primiparous so the majority of participants were able to contrast their experience during this pregnancy to previous pregnancies prior to the pandemic. It is important to give due consideration to the specific challenges faced by ethnic minority women during this period such as exacerbation of communication issues and increased barriers to accessing necessary care.

Conclusion

This study provides insight into the specific challenges faced by ethnic minority women in pregnancy, which intersect with the unique problems posed by the ongoing SARS-CoV-2 pandemic to potentially widen existing ethnic disparities in maternal outcomes and experiences of maternity care.

Keywords

Race; Ethnic minority; Pregnancy; SARS-CoV-2; Qualitative research; maternity services

Contribution to authorship

JJ was responsible for the conception of the study, planning, delivery, qualitative interviews, analysis of the study and wrote the first draft of the paper. GC and SCB contributed to the planning of the study, interpretation of results and provided critical feedback on the draft paper. All authors read and approved the final manuscript

Disclosure of interests

None declared. Completed disclosure of interests forms are available to view online as supporting information.

Details of ethics approval

The study was approved by the Research Ethics Committee of West of Scotland (20/WS/0168).

Funding

NIL to declare.

Acknowledgements

We would like to thank all of the women that participated in this study and acknowledge the help of obstetricians and midwives who assisted with recruitment.

References

1- World Health Organization; Maternal, newborn, child and adolescent health: Maternal and perinatal death reviews in the UK. Accessed 08/02/2021.

2- Knight M, Bunch K, Tuffnell D et al on behalf of MBRRACE-UK. Saving Lives, Improving Mothers' Care - Lessons learned to inform maternity care from the UK and Ireland Confidential Enquiries into Maternal Deaths and Morbidity 2016-18. Oxford: National Perinatal Epidemiology Unit, University of Oxford 2020.

3- D'Souza L, Garcia J: Improving services for disadvantaged childbearing women. Child Care Health Dev. 2004, 30 (6): 599-611.

4- Knight Marian, Bunch Kathryn, Vousden Nicola et al. Characteristics and outcomes of pregnant women admitted to hospital with confirmed SARS-CoV-2 infection in UK: national population based cohort study BMJ 2020; 369 :m2107

5- Nowell LS, Norris JM, White DE, Moules NJ. Thematic Analysis: Striving to Meet the Trustworthiness Criteria. International Journal of Qualitative Methods. December 2017.

6- Edwards , N.P. , 2004 . Why can't women just say no? And does it really matter? In : M. Kirkham Informed choice in maternity care . Basingstoke : Palgrave MacMillan , 1 – 22 .

7- Ockleford, E.M., Berryman, J.C. and Hsu, R. 2004. Postnatal care: what new mothers say. British Journal of Midwifery, 12(3): 166–170.

8- Jomeen, J. 2010. Choice, control and contemporary childbirth: understanding through women's experiences, Oxford: Radcliffe.

9- Office for National Statistics, U.K.: Immigration, 1990. Accessed 08/02/2021

10- Office for National Statistics, U.K.: Immigration 2019. Accessed 08/02/2021

11- Deborah Turnbull, Mary McGinley, Gillian Mcllwaine, Ian A. Greer, Changing childbirth, I and II, BJOG: An International Journal of Obstetrics & Gynaecology, 10.1111/j.1471-0528.1994.tb11966.x

12- Equality Act 2010 (Commencement No.3) Order 2010.

13- World Health Organisation, Gender, equity and human rights: The right health, Factsheet No. 31, Office of the United Nations High Commissioner for Human Rights. Accessed 08/02/2021

14- McCourt, C. and Pearce, A. 2000. Does continuity of carer matter to women from minority ethnic groups?. Midwifery, 16: 145–154.

15- Cross-Sudworth, F., Williams, A. and Herron-Marx, S. 2011. Maternity services in multi-cultural Britain: using Q methodology to explore the views of first and second generation women of Pakistani origin. Midwifery, 27(4): 458–468.

16- Julie Jomeen & Maggie Redshaw (2013) Ethnic minority women's experience of maternity services in England, Ethnicity & Health, 18:3, 280-296

17- Lyons, S.M. 2008. Cultural diversity in the Dublin maternity services: the experience of maternity service providers when caring for ethnic minority women. Ethnicity and Health, 13(3): 261–276.

	N (%)
**Ethnicity**	
Black-African	7 (43.7)
Black-Caribbean	1 (6.3)
Asian-Indian	3 (18.7)
Asian-Chinese	1 (6.3)
Asian-Bangladeshi	1 (6.3)
Asian-Pakistani	1 (6.3)
Arab	2 (12.5)
**Age**	
25 – 29	1 (6.3)
30 – 34	10 (62.5)
35 – 40	4 (24.9)
>41	1 (6.3)
**Religion**	
Christian	6 (37.5)
Muslim	7 (43.7)
Hindu	1 (6.3)
Atheist	2 (12.5)
**Country of birth**	
U.K.	1 (6.2)
Outside U.K.	15 (93.8)
**Year of immigration**	
N/A as born within U.K.	1 (6.3)
2000 - 2009	3 (18.7)
2010 - 2019	11 (68.7)
2020	1 (6.3)
**Parity**	
Nulliparous	2 (12.5)
One	6 (37.5)
Two	6 (37.5)
Three	2 (12.5)
**Antenatal/Postnatal**	
Antenatal	9 (56.3)
Postnatal	7 (43.7)
**Comorbidities**	
Yes	2 (12.5)
No	14 (87.5)
**First languages**	
Fulani	2 (12.5)
Yoruba	2 (12.5)
Arabic	1 (6.3)
Bengali	1 (6.3)
English	1 (6.3)
Farsi	1 (6.3)
French	1 (6.3)
Hindi	1 (6.3)
Igala	1 (6.3)

For peer review only

**Communication**
Respect
P1 I never feel like I am not in my country.<br>P6 I could sense that she was busy and she could have rather been off, you know, not having that one extra person popping into triage at that point. For me, it was just a case of, well, you know, I need this, so I can only apologise, but there's nowhere else I can go.<br>P11 I will also want some respect as well, because I'm, I'm capable to getting their respect. So, if other people win respect then I have to make the confidence well yes, I'm capable of getting their respect.<br>P15 I had a CVS at eleven weeks and during the procedure, I was kind of, um, emotional. Um, I was being talked to by the nurses and the midwives very well. I really appreciated the way they spoke to me.
Accent bias
P12 I don't call about everything. But when you have to call through, you're not taken seriously once your accent is heard. That's just the truth, yeah. I just feel that the…there…there's a little bit of language when it comes to…once your accent is heard, you…you…you are not the privileged lot, let me put it that way. So nobody's gonna appoint you an appointment. They don't…they don't think it's necessary
Language barrier
P8 So, therefore, if I'm given the same sort of information at the same level…with someone that is English speaking, the person that is English speaking will understand a lot better…and probably make better decisions than I would.<br>P2 It creates like a…a gap in communication where if something you express is not clearly understood so maybe they could be left with some misinterpretation<br>P9 Um, you know, when I initially came my English was not good. Eh, and it was harsh for me, the…with the way they communicated…because I don't know the language and they know; they know that I don't know the language. And I'm already depressed because I don't know whether my baby will survive or not.<br>P14 So if she feels confident to speak about the baby, to phone and speak about the issue, she, she will…yeah, she will do it. But if she feels that the person will not…she's phoning will not understand her because she won't be able to deal with the message or to say whatever she want to say, she prefers not to phone and to explore other solutions. It's really difficult sometimes…eh, to know what to do or who to speak to.
Cultural dissonance
P2 how your culture is too…can…can be a factor as well. 'Cause, umm, this can…this can cause a disparity where…a misinterpretation again where…where in my culture this is okay…But…and…and how I express myself, it might come off as you're

Jamaican Patois	1 (6.3)
Mandarin	1 (6.3)
Spanish	1 (6.3)
Telagu	1 (6.3)
Urdu	1 (6.3)
**Employment**	
Yes	13 (81.3)
No	3 (18.7)

Table 1: Study participant characteristics

For peer review only

not being accepted but, umm, it doesn't mean…it does not come off from a negativity on my side 'cause it's…it's my culture……but because someone else is from a different culture and they clash, there…there comes a…a misinterpretation, so that can affect care as well<br>P5 Um, and then the circumcision part of it is just on hold at the moment. Um, and, and that's…that's something that's quite important for us to get kind of done straightaway<br>P8 …and as a Nigerian I think people just think, a Caesarean section, no no no no,…because that has happened. So the first…the first thing I had when I have a planned C-section like, oh my gosh, have you tried? Don't you know God can heal you?<br>P11 One thing people, sometimes I find some people don't like, so well, I don't want to show people that I'm Muslim. There are lots of times because of Muslim, because of the bomb blast and other sorts of things, they are pointing me.

Table 2: Direct quotations relating to theme of communication

For peer review only

**Interactions with health care professionals**
Continuity of care
P2 So the continuity of care both in and outside of appointments, so it was just quite tremendous, just really good. …that has impressed me as well, and, umm, it's…it's just the thoroughness of it, to be honest, so your journey is not left at the hospital.<br>P3 And then every time a different doctor would come with a different approach……and they were not really clear and explaining to me what's going on, so I was very stressed. Um, and I really wanted to see a consultant, but I had to wait for that for quite a long time. Kind of I had to cry actually to. And I wasn't relying on the system anymore because I was hearing different opinions every time.<br>P7 So maybe they thought that I was still seen by my midwives in my, in my GP surgery or something, and they were…they never checked the size of your, um, tummy and they did not really check the position of the baby and all those kind of things.
Empathy
P3…and the midwives were very busy, um, so they couldn't attend me. And at one point, I just asked the midwife to, to look after my baby while I go to the toilet, and when I came back, she was not there and my baby had, like, vomited all over her face. So it was really, really the worst time of my life<br>P9 But the only thing what I felt was they haven't paid enough…much empathy for a human being. Eh, what I felt most was they didn't care enough to care for a human being. It was just like a product, that's what I felt. I was just like a product over there. There was no value for the emotions.<br>P12 I had a horrible…horrible experience with the…the midwives…midwives on…on duty. Okay, I've had two…I had two kids but I had no complication and so having the third one and I was, err…I…I had to go through an emergency Caesarean and all that it was very traumatic for me as a person and…but I don't think they understood that.
Informed decision making
P3 but it was mainly up to me as well to read and ask questions…rather than getting, um, like information.<br>P6 I've been given the Ready, Steady, Baby book and stuff…so there is reading materials and links and online antenatal classes, but I wouldn't say there's been that much support in terms of, you know, actually sitting me down and talking to me and explaining different things, it's been more, this is where you can go and read on stuff if you want.
Dissonance with other health care systems

P3 I would go and I would come back and she was shocked every time because the process in Iran is really different. I was wishing that I was actually in Iran so I could get more help from specialists.<br>P12 As much as you have to pay to get, umm, your baby delivered in America…they…they have a lot more care than in the United Kingdom.<br>P14 In Jordan she used to go every month to the doctor to check everything about the baby. She really wish if, whenever she goes to see the midwife, there is something to assure her about the baby's health…

Table 3: Direct quotations relating to theme of interaction with health care professionals

For peer review only

**Racism**
Institutional
P8 Treatment for black people will be different from treatment for white people.<br>P10 Um, they are white, they don't want to talk to you.<br>P12 Most of the time for me to get care for my children, I have to say it with a very loud voice or sounding like the child is just they're going to die in a minute before I'm responded to.
Interpersonal
P6 Um, and she said that no, this is not right, you know, um, she'd kind of gone to the GP and stuff, and she'd spoken to the midwives and they were like, oh, it's nothing that serious, but at the same time, there was white women who were having the same issue, but being taken more seriously upon.<br>P10 Yeah, because they didn't listen to us anyway. I think they listen to their people than, you know, us, and they didn't have the patience. Because I remember when I had my baby, and then I had a C-section when I was in the hospital, I was so much in pain when I called one of the midwives to come and help me to feed my baby, she was proper shouting, oh, you need to try it for yourself, you know. Just not…just not being polite. Talking to me, you know, so rude.<br>P12 in the United Kingdom they act as if you are privileged to be…so I'm…I'm also talking as, err, B…BAME, right? Like a black woman, right? They act like you're privileged that you are not having a baby in…in the bush.
Internalised
P6 so I've got a bit more I guess, a cold-headed, um, kind of, I guess, but I'm quite direct, and it's only made me, like hearing all these cases and stories and, you know, the women's experience, it only kind of makes me a bit more, I guess, hard skinned, um, just to make sure that, you know, I need to make sure that no-one is overlooking me, just for the sake of my skin.<br>P7 Well, I believe that we receive equal healthcare regardless of the colour of the skin. But what I think, the body, the nature of the body or the gene is different in compared to the people here. Our gene or the nature of our body and whatever it is quite different… Um, I, I think our bodies are much more weak or something like that maybe. Maybe less immune or less resistant, or maybe like that.<br>P11 It's not just only country, my culture, my, my view, not only religion, because we are third world country…I'm not from the developed country. When…if I'm coming from like Canada or the US or something I can, I can show that confidence

Table 4: Direct quotations relating to theme of racism

**The pandemic effect**
Isolation
P2 So COVID kind of, umm, robbed me of that experience which I'm used to back at home, where I used to attend my clinics…So clinics gave you that…that…that, umm, avenue of socialisation, meeting another mothers, new mothers……creating, umm, friendships…and…and also, you know, a continuance after birth<br>P3 I'm not getting enough support for taking care of my baby, she's crying day and night, and I couldn't have my husband or anyone because of COVID<br>P5 I had to go to all my appointments on my own.
Psychological impact
P11 COVID actually is really stressful mentally…you know. I can't go anywhere, nobody can come, which is in a pregnant woman, uh, isn't…I'd love to go somewhere, but we couldn't go anywhere because of the restriction.<br>P13 I found it very hard when you're coming to the country without knowing anyone and the coronavirus, everyone is…doing lockdown so it was very…difficult, I was very depressed. I was very anxious, yeah, umm, I feel worried a lot.
Barriers to access of care
P5 I think there were disadvantages, you don't have that face to face contact.<br>P6 I don't know if that's the pandemic causing people to be a bit more relaxed and, you know, like okay, here's the leaflet, you can go and do the reading.<br>P10 Because when I was explaining about my breathing, they didn't want to see me, they were saying that they think that I'm having a coronavirus…and I really forced, yeah, I said I'm not having a coronavirus, it's like this is… I know that this is related to my cardiomyopathy. I have really, really struggled, I can say that. I've been calling, calling, talking, talking, they don't want to see me.<br>P11 But they said, no you can't come, you have to send a picture and you have to email us. Which is sometimes, you know, is not easy to do with the phone. Because I'm not a doctor.<br>P14 she can't phone the GP to describe what is the problem. And because of the Corona, she understands that it's not easy to get an appointment. So she tried to read on the internet how she can help herself.

Table 5: Direct quotations relating to the pandemic effect

For peer review only

For peer review only

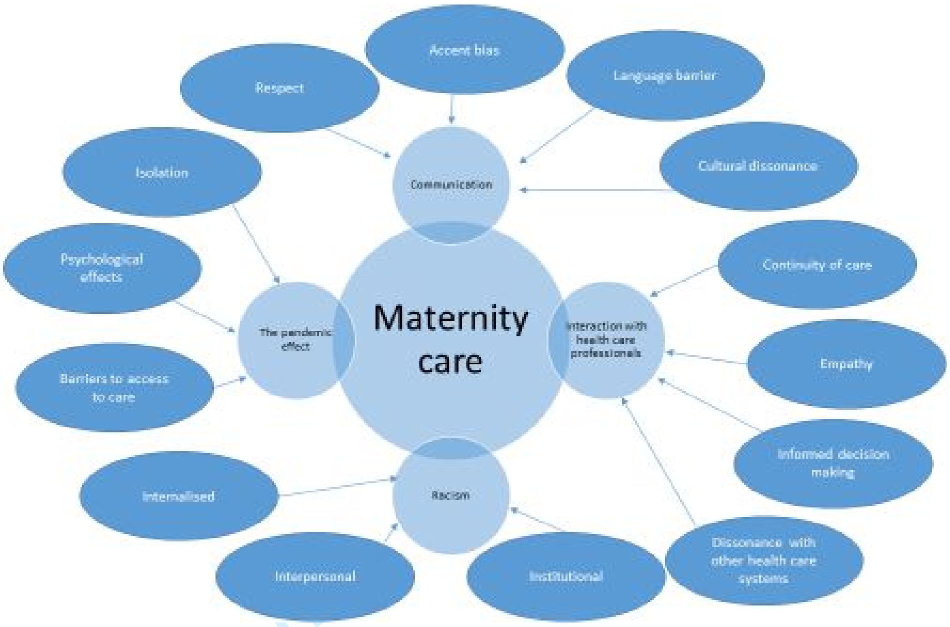

Figure 1: Themes and Subthemes

For peer review only

Qualitative interview topic guide

Interviews with pregnant women will be semi structured in nature but emphasis will be given to 3 main topics of:

1. Communication,
2. Access to maternity health care,
3. Cultural dissonance

Examples of questions under each sub heading will be described below:

1. Communication:
    - Are you able to communicate effectively and easily with your maternity health care providers (such as midwife/ obstetrician)?
    - Do you feel that your maternity health care providers are able to provide you with all the information and support you require in this pregnancy?
    - (Where indicated) are NHS interpreters that you have encountered so far able to answer the questions you have had relating to your care?

2. Access to maternity health care:
    - Are there any barriers to access maternity health care that you have come across especially relating to telephone triage?
    - If yes, how do you think this could be improved?
    - Have you felt that your concerns have not been listened to in this pregnancy?
    - Do you feel adequately supported if any concerns were to arise in this pregnancy?

3. Cultural dissonance:
    - Have you previously had maternity care elsewhere?
    - During this pregnancy, have you sought advice regarding health care from health care professionals outside the U.K.?
    - Do you feel that everyone in the U.K. receives equal health care regardless of their race? If not, do you have personal examples of bias in treatment or racism in health care?

Other questions that are relevant but do not fit into the themes above include:

- What are the beliefs, concerns, and expectations in relation to health of BAME women in pregnancy?
- What are their experiences of maternity care at different stages of pregnancy?
- How do women feel about being pregnant as a risk in the context of COVID-19?
- What do BAME women see as the main challenges to ensuring equality in maternity care?

For peer review only

Consolidated criteria for reporting qualitative studies (COREQ): 32-item checklist

Developed from:
Tong A, Sainsbury P, Craig J. Consolidated criteria for reporting qualitative research (COREQ): a 32-item checklist for interviews and focus groups. *International Journal for Quality in Health Care*. 2007. Volume 19, Number 6: pp. 349 – 357

YOU MUST PROVIDE A RESPONSE FOR ALL ITEMS. ENTER N/A IF NOT APPLICABLE

No. Item	Guide questions/description	Reported on Page #
**Domain 1: Research team and reflexivity**		
Personal Characteristics		
1. Inter viewer/facilitator	Which author/s conducted the interview or focus group?	Methods (Page 3)
2. Credentials	What were the researcher's credentials? E.g. PhD, MD	Introduction (Page 1)
3. Occupation	What was their occupation at the time of the study?	Discussion (Page 8)
4. Gender	Was the researcher male or female?	Female
5. Experience and training	What experience or training did the researcher have?	Qualitative research methodology training through University of Edinburgh
Relationship with participants		
6. Relationship established	Was a relationship established prior to study commencement?	Discussion (Page 8)
7. Participant knowledge of the interviewer	What did the participants know about the researcher? e.g. personal goals, reasons for doing the research	Discussion (Page 8)
8. Interviewer characteristics	What characteristics were reported about the inter viewer/facilitator? e.g. Bias, assumptions, reasons and interests in the research topic	Discussion (Page 8)
**Domain 2: study design**		
Theoretical framework		
9. Methodological orientation and Theory	What methodological orientation was stated to underpin the study? e.g. grounded theory, discourse analysis, ethnography, phenomenology, content analysis	Methods (Page 4)
Participant selection		
10. Sampling	How were participants selected? e.g. purposive, convenience, consecutive,	Methods (Page 3)

For peer review only

	snowball	
11. Method of approach	How were participants approached? e.g. face-to-face, telephone, mail, email	Methods (Page 3)
12. Sample size	How many participants were in the study?	Results (Page 4)
13. Non-participation	How many people refused to participate or dropped out? Reasons?	N/A
Setting		
14. Setting of data collection	Where was the data collected? e.g. home, clinic, workplace	Methods (Page 3)
15. Presence of non-participants	Was anyone else present besides the participants and researchers?	Methods (Page 4)
16. Description of sample	What are the important characteristics of the sample? e.g. demographic data, date	Table 1: Study participant characteristics)
Data collection		
17. Interview guide	Were questions, prompts, guides provided by the authors? Was it pilot tested?	Interview topic guide
18. Repeat interviews	Were repeat inter views carried out? If yes, how many?	No
19. Audio/visual recording	Did the research use audio or visual recording to collect the data?	Yes
20. Field notes	Were field notes made during and/or after the inter view or focus group?	During
21. Duration	What was the duration of the inter views or focus group?	30 mins – 1 hour
22. Data saturation	Was data saturation discussed?	Yes
23. Transcripts returned	Were transcripts returned to participants for comment and/or correction?	No
**Domain 3: analysis and findings**		
Data analysis		
24. Number of data coders	How many data coders coded the data?	1
25. Description of the coding tree	Did authors provide a description of the coding tree?	Yes
26. Derivation of themes	Were themes identified in advance or derived from the data?	Derived from data
27. Software	What software, if applicable, was used to manage the data?	MS Word
28. Participant checking	Did participants provide feedback on the findings?	No
Reporting		
29. Quotations presented	Were participant quotations presented to illustrate the themes/findings? Was each quotation identified? e.g. participant number	Yes
30. Data and findings consistent	Was there consistency between the data presented and the findings?	Yes
31. Clarity of major themes	Were major themes clearly presented in the findings?	Yes
32. Clarity of minor themes	Is there a description of diverse cases or	Yes

For peer review only

	discussion of minor themes?	

Once you have completed this checklist, please save a copy and upload it as part of your submission. When requested to do so as part of the upload process, please select the file type: *Checklist*. You will NOT be able to proceed with submission unless the checklist has been uploaded. Please DO NOT include this checklist as part of the main manuscript document. It must be uploaded as a separate file.

For peer review only

BMJ Open

Exploring ethnic minority women's experiences of maternity care during the SARS-CoV-2 pandemic: a qualitative study

Journal:	*BMJ Open*
Manuscript ID	bmjopen-2021-050666.R1
Article Type:	Original research
Date Submitted by the Author:	12-Jun-2021
Complete List of Authors:	John, Jeeva; The University of Edinburgh Usher Institute of Population Health Sciences and Informatics,<br>Curry, Gwenetta; The University of Edinburgh Usher Institute of Population Health Sciences and Informatics<br>Cunningham-Burley, Sarah; The University of Edinburgh Usher Institute of Population Health Sciences and Informatics, Public Health Sciences
**Primary Subject Heading**:	Obstetrics and gynaecology
Secondary Subject Heading:	Health services research, Patient-centred medicine, Qualitative research
Keywords:	QUALITATIVE RESEARCH, COVID-19, HEALTH SERVICES ADMINISTRATION & MANAGEMENT, Organisation of health services < HEALTH SERVICES ADMINISTRATION & MANAGEMENT, Quality in health care < HEALTH SERVICES ADMINISTRATION & MANAGEMENT, OBSTETRICS

SCHOLARONE™
Manuscripts

I, the Submitting Author has the right to grant and does grant on behalf of all authors of the Work (as defined in the below author licence), an exclusive licence and/or a non-exclusive licence for contributions from authors who are: i) UK Crown employees; ii) where BMJ has agreed a CC-BY licence shall apply, and/or iii) in accordance with the terms applicable for US Federal Government officers or employees acting as part of their official duties; on a worldwide, perpetual, irrevocable, royalty-free basis to BMJ Publishing Group Ltd ("BMJ") its licensees and where the relevant Journal is co-owned by BMJ to the co-owners of the Journal, to publish the Work in this journal and any other BMJ products and to exploit all rights, as set out in our licence.

The Submitting Author accepts and understands that any supply made under these terms is made by BMJ to the Submitting Author unless you are acting as an employee on behalf of your employer or a postgraduate student of an affiliated institution which is paying any applicable article publishing charge ("APC") for Open Access articles. Where the Submitting Author wishes to make the Work available on an Open Access basis (and intends to pay the relevant APC), the terms of reuse of such Open Access shall be governed by a Creative Commons licence – details of these licences and which Creative Commons licence will apply to this Work are set out in our licence referred to above.

Other than as permitted in any relevant BMJ Author's Self Archiving Policies, I confirm this Work has not been accepted for publication elsewhere, is not being considered for publication elsewhere and does not duplicate material already published. I confirm all authors consent to publication of this Work and authorise the granting of this licence.

For peer review only

Exploring ethnic minority women's experiences of maternity care during the SARS-CoV-2 pandemic: a qualitative study

Dr. Jeeva John MBChB[1] (Corresponding author: jeeva.john@ed.ac.uk, Ph: 07792562199) Dr. Gwenetta Curry[2], Prof. Sarah Cunningham-Burley[1]

1- The University of Edinburgh Usher Institute of Population Health Sciences and Informatics, Centre for Biomedicine, Self and Society, Edinburgh, Edinburgh, UK

2- The University of Edinburgh Usher Institute of Population Health Sciences and Informatics, Usher Institute, Edinburgh, Edinburgh, UK

Running title: Ethnic minority maternity care during Covid-19

For peer review only

Abstract

Objective:

To explore the experiences of pregnancy, childbirth, antenatal and postnatal care in women belonging to ethnic minorities and to identify any specific challenges that these women faced during the SARS-CoV-2 pandemic.

Design:

This was a qualitative study using semi structured interviews of pregnant women or those who were six weeks postnatal from Black, Asian, and Minority Ethnic backgrounds. The study included sixteen women in a predominantly urban Scottish health board area.

Results

The finding are presented in four themes: ‘communication’, ‘interactions with health care professionals’, ‘racism’ and ‘the pandemic effect’. Each theme had relevant sub-themes. ‘Communication’ encompassed respect, accent bias, language barrier and cultural dissonance; ‘interactions with health care professionals’: continuity of care, empathy, informed decision making and dissonance with other health care systems; ‘racism’ was deemed to be institutional, interpersonal or internalised; and ‘the pandemic effect’ consisted of isolation, psychological impact and barriers to access of care.

Conclusions

This study provides insight into the specific challenges faced by ethnic minority women in pregnancy, which intersect with the unique problems posed by the ongoing SARS-CoV-2 pandemic to potentially widen existing ethnic disparities in maternal outcomes and experiences of maternity care.

Funding:

Ethical approval for this study was granted by the Research Ethics Committee of West of Scotland (20/WS/0168) on 26/11/2020.

For peer review only

Strengths and limitations of this study

- This study addresses a gap in the literature by using qualitative methods to provide an in-depth exploration of minority ethnic women's experiences of maternity care during the SARS-CoV-2 pandemic.
- This study explores the perspectives of pregnant ethnic minority women at different stages of pregnancy, as well as the postnatal period.
- A flexible topic guide was developed using existing literature, in collaboration with an obstetrician, a social scientist and a race and ethnicity lecturer, bringing together different ideologies to a complex issue.
- The interviewer is an obstetrician, and although none of the participants were known to the interviewer clinically, they were aware of the interviewer's role, and it is important to acknowledge that this could have had an indirect effect on participant responses.

For peer review only

Introduction

Whilst the overall Maternal Mortality Rate (MMR) in the U.K. has remained relatively low over the past decade[1], the gulf in maternal outcomes between White women and women from ethnic minority backgrounds continues to expand. The latest U.K. confidential enquiry into maternal deaths (MBRRACE-U.K) report showed significant racial variations in maternal mortality. Black women were four times, while Asian women were two times as likely to die as White women during pregnancy, delivery or postpartum between 2016 and 2018.[2] These results are comparable to the MMR in minoritised ethnic groups in the U.S.A and Netherlands despite key differences in health care systems.[3.4] Inequalities in maternal outcomes are long standing and reproducible in other high-income countries, however the causes of these disparities remain unclear. Ethnic minorities form a significant proportion of some of the most disadvantaged groups in the U.K. facing intersecting social determinants of health including unemployment and deprivation.[5,6] Although some studies have demonstrated correlation between these social elements and poor maternal outcomes, they do not fully explain the gap.[7,8] Previous studies have investigated the experiences of pregnant immigrant women accessing maternal care in the U.K. Women who had negative experiences with health care professionals avoided accessing maternal services.[9] Unsurprisingly, not engaging with maternal care services can negatively impact both maternal and foetal health thereby increasing the risk of severe morbidity and mortality. On the contrary, women from ethnic minority backgrounds who did wish to engage, experienced limitations in accessing maternal care services in the UK, which also led to poorer health outcomes.[10] In country wide surveys of maternity care, ethnic minority women were more likely to report poor patient experience; in some cases, attributed to stereotyping and racism.[11,12] Experiences of racism and discrimination have also been linked to poor outcomes for minoritised populations.[13] The SARS-CoV-2 pandemic has not only shone a spotlight on these disparities but may have exacerbated them.[14] A key component in establishing equality of maternal healthcare provision, is the examination of women's experiences of these services. There is little qualitative research on the lived experiences of all ethnic minority women during or immediately following pregnancy in the U.K. Previous qualitative literature in the U.K. has tended to focus on nuanced aspects of maternal care, [15] particular health conditions during pregnancy, [16] or specific ethnic groups.[17] This study aimed to explore the experiences of pregnancy, childbirth, antenatal and postnatal care in all women belonging to ethnic minority communities and to identify any specific challenges that these women faced during the SARS-CoV-2 pandemic.

For peer review only

Methods

[illegible]

Participant Characteristics and Recruitment

Women who were pregnant or within six months of delivery from a Black, Asian, and Minority ethnic background were recruited for the current study. Sixteen women were recruited: 7 Black African, 1 Black Caribbean, 3 Asian-Indian, 1 Asian-Chinese, 1 Asian-Bangladeshi, 1 Asian-Pakistani, and 2 Arab. Nine of the women were antenatal and seven were postnatal. All but one of the participants had themselves been born outside the U.K.

This study was performed in all of the community midwifery hubs and obstetric units within a predominantly urban Scottish health board. A health board is a regional authority in Scotland with local responsibility for the delivery of health services. During the SARS-CoV-2 pandemic, changes to delivery of maternal health care within this health board followed national and Royal College of Obstetricians and Gynaecologists' guidance, and included a universal shift from face-to-face consultations to telephone clinics, and restriction of visitors during hospital admission during scan appointments and following delivery.[18] Units were provided with study recruitment packs containing a participant information sheet, consent form and pre-paid envelope. Midwives were encouraged to share study information with ethnic minority patients and to provide them with recruitment packs. With their consent, participant details were securely passed onto researcher, JJ, who telephoned each patient 48 hours after receiving a recruitment pack. Participants who could not speak English were provided appropriate translations of study documents and interpreters were made available for interviews. All participants confirmed consent prior to each interview. The interview topic guide was developed by all three authors and was used to aid semi-structured interviews carried out by JJ.

For peer review only

Data Collection

Data collection occurred between December 2020 and January 2021. Audio-recorded interviews were transcribed verbatim by First Class Secretarial Services. Collected data was kept confidential by allocating a distinct code to each woman to protect anonymity. Data was collected until no new themes were identified and inductive and deductive thematic saturation was achieved.

Data Analysis

This study adopted a data analysis methodology based on thematic analysis.[19] The transcriptions were read and re-read, a coding frame was constructed, and the data coded to identify initial themes. A qualitative interpretive approach was taken and thematic analysis was conducted with continuous consultation between researchers JJ and GC. A consensus discussion regarding theme development was undertaken by all three authors.

Patient and public involvement

There was no formal PPI group that assisted with this study. However, in the course of recruitment and interviews, advice from participants and health care professionals was sought and used to create a flexible interview topic guide. A summary of study findings will be available on the Centre for Biomedicine, Self and Society for general access, and study findings will also be disseminated amongst study participants with consent having been obtained at time of interview.

Details of ethics approval

Ethical approval for this study was granted by the Research Ethics Committee of West of Scotland (20/WS/0168).

Results

Sixteen women participated in telephone interviews (see Table 1 for sample characteristics).

Four principal themes were identified from interview data. These included communication, interactions with healthcare professionals, racism, and the pandemic effect. Each theme had subthemes, as presented in Figure 1.

Direct quotes relating to each theme are presented in Tables 2, 3, 4 and 5.

Communication

Respect

Participants who were satisfied with the maternal health system made reference to respectful communication. This was particularly important during episodes of high maternal stress (Table 2: *Respect,* P15). Participants concurred that community midwives and health visitors were considerate in their routine communication (Table 2: *Respect,* P1). A significant majority of the group conversely felt that they received disrespectful care during unsolicited hospital visits (Table 2: *Respect,* P11; P16).

Accent bias

All participants except one were first-generation immigrants with median duration of stay in the U.K. of nine years. The majority of the cohort had non "British" accents and identified bias due to accent as being a significant concern, sometimes perceived to impede access to emergency care, and prevent equality of maternal care received. One participant highlighted having a non "Westernised" accent as being interpreted as a proxy for lower socio-economic status and educational attainment, and that this was a unique barrier with regards to telephone consultations (Table 2: *Accent bias*, P12).

For peer review only

Language barrier

In this study, most participants spoke English fluently; only one required an interpreter. Despite the high standard of English spoken, most participants felt that language barriers were the most common cause of miscommunication between themselves and healthcare professionals. They concurrently felt they themselves were more likely to make inappropriate decisions regarding their healthcare as a result of misinterpretation (Table 2: *Language barrier*, P8). Healthcare professional misunderstanding due to language barriers was also described as a prime feature in barriers to effective communication (Table 2: *Language barrier*, P2; P9). In contrast, the participant who required an Arabic interpreter had not experienced any instances of miscommunication when an interpreter was present during pre-organised appointments. However, she admitted facing major communication challenges during unscheduled calls when an interpreter was not available. She identified a gap in recognition from healthcare professionals regarding this particular scenario, and reported that she often resorts to searching the internet for pregnancy advice even during emergencies due to anxiety surrounding communication (Table 2: *Language barrier*, P14).

Cultural dissonance

Cultural dissonance was identified as another significant barrier to effective communication. Cultural dissonance between participant and healthcare professional (Table 2: *Cultural dissonance*, P2) as well as between participants and their wider community (Table 2: *Cultural* dissonance, P8) both impacted quality of communication equally. This could be in the form of religious discordance in routine clinical practice (Table 2: *Cultural* dissonance, P11) or misunderstanding of wider cultural context (Table 2: *Cultural dissonance*, P5).

Interactions with healthcare professionals

Quality of interaction with healthcare professionals was generally associated with four key domains: continuity of care; empathy; informed decision making; and dissonance with previous experiences of maternity care. This theme, although strongly interlinked with the previous one, was derived separately to emphasise the non-verbal, and institutional aspects of health care interactions that affected participants' experiences of maternity care.

Continuity of care

Overall, participants felt they received good continuity of care throughout their pregnancy. Primiparous women particularly valued routine postnatal check-ups (Table 3: *Continuity of care*, P2). A common sentiment that arose in women requiring regular input from secondary care during the antenatal period was ineffective communication between their community midwives and hospital midwives or obstetricians, and vice-versa, sometimes resulting in omission of crucial clinical

For peer review only

information (Table 3: *Continuity of care*, P7). In the women who experienced prolonged hospital admissions, the inability to discuss ongoing care with the same healthcare professionals led to varying information being given to the patient leading to dissolution of trust (Table 3: *Continuity of care*, P3).

Empathy

The most important aspect of interactions with healthcare professionals was the presence of empathy. In instances where the healthcare professional was deemed indifferent, participants often cited being hurried, feeling unheard, and uncared for, which negatively impacted the interaction, and the overall perception of maternity care (Table 3: *Empathy*, P3). Participants who experienced apathy in previous pregnancies recognised that the possibility of recurrence was a source of anxiety throughout subsequent pregnancies (Table 3: *Empathy*, P9; P12).

Informed decision making

Although all participants reported some level of information provision from healthcare providers regarding clinical decision making, almost everyone agreed that they would benefit from more thorough discussions. Most participants received information about their pregnancy in the form of signposting to books or websites but they expressed that their individual information needs would have been better met by one-to-one discussions (Table 3: *Informed decision making*: P6). Women who felt that their questions remained unanswered did not feel involved in shared decision making (Table 3: *Informed decision making:* P3).

Dissonance with other healthcare systems

Fifty percent of the study population had experienced maternity care out with the present health board, with a significant proportion having delivered outside the U.K. All participants who previously delivered outside the U.K. had experienced privatised healthcare.

Access to early pregnancy care, and shared decision making were some of the reasons provided by women who previously delivered in other parts of the U.K. reporting higher satisfaction with the study health board in the current pregnancy. The majority of the participants who had delivered abroad felt that their previous pregnancy experiences were generally better compared to care received in this health board. Reasons given for this discordance varied amongst individual participants, and included; improved access to ultrasound scans, availability of medical specialists, and treatment by healthcare providers (Table 3: *Dissonance with other healthcare systems*, P12; P14). A high proportion of primiparous women had sought advice from relatives, friends and/or healthcare professionals in their country of birth (Table 3: *Dissonance with other healthcare systems*, P3).

For peer review only

Racism

Institutional

Institutional racism was highlighted as a significant issue in pregnancy care by most of the participants. A small proportion of Black participants concomitantly worked in healthcare, and were able to provide examples of racial discrimination against themselves from their colleagues (Table 4: *Institutional*, P10). Some of the participants expressed distrust in maternal healthcare due to their concerns that medical research and treatments are tailored for their White counterparts (Table 4: *Institutional*, P8). Participants who had experienced institutional racism were also likely to perceive barriers to accessing healthcare compared to White women (Table 4: *Institutional*, P12).

Interpersonal

Most women gave examples of acts of racism against their friends, or family rather than themselves (Table 4: *Interpersonal*, P6; P12).

Internalised

In participants who expressed internalised racist beliefs, these almost always affected health behaviours. In one example, a significant delay in diagnosis of a common condition led one participant to believe that the main problem was her lack of assertiveness compared to White women (Table 4: *Internalised*, P6). In another case, a participant felt that she did not have the right to question the healthcare she was receiving, being originally from a low-income country (Table 4: *Internalised,* P11). Yet another participant strongly believed that disparities in maternal health outcomes could be explained by physiological differences between ethnic groups (Table 4: *Internalised*, P7).

The pandemic effect

This study was developed in order to better understand the lived experiences of women from ethnic minority backgrounds during maternity care. Due to the time period that the study was conducted in, the SARS-CoV-2 pandemic and its' direct and indirect consequences were naturally brought up by all participants. The sub-themes below illustrate the most common impacts of the pandemic as mentioned by the participants.

Isolation

Most women reported feeling isolated during their pregnancy due to features specific to the SARS-CoV-2 pandemic. This was particularly a problem for those who felt that they would have benefitted from the presence of a companion when important information relating to their pregnancy was being relayed to them (Table 5: *Isolation*, P5). Although most women were understanding of the limitations posed by the pandemic, a significant proportion expressed loneliness exacerbated by not being able to

For peer review only - http://bmjopen.bmj.com/site/about/guidelines.xhtml

engage with their usual pregnancy support networks, and did not feel that virtual groups mitigated this effect (Table 5: *Isolation,* P2; P3).

Psychological impact

The detrimental effect of the pandemic on mental health during pregnancy was highlighted by all participants. Women expressed feeling more anxious, fearful, and lacking autonomy in comparison to their previous pregnancies. This sentiment was particularly emphasized by the participants who had little or no family members nearby (Table 5: *Psychological impact*, P11; P13). The majority of participants reported that they were not routinely asked about their mental health in relation to the pandemic by healthcare professionals. Fewer still were signposted to appropriate support groups for help.

Barriers to access of care

Most participants had some of their routine face-to-face appointments replaced by telephone calls. The majority felt that virtual appointments were not as effective, especially during unscheduled care (Table 5: *Barriers to access of care*, P5). Women expressed uncertainty surrounding the accuracy of information relayed via telephone. (Table 5: *Barriers to access of care*, P6). In circumstances where participants did seek physical consultations, they experienced barriers, and often had to repeatedly call in order to be seen (Table 5: *Barriers to access of care*, P10; P11). On the contrary, some women delayed seeking medical help due to apprehensions surrounding contracting SARS-CoV-2 (Table 5: *Barriers to access of care*, P14).

Discussion

Main findings

This study focussed on the lived experiences during the SARS-CoV-2 pandemic of minority ethnic women who were pregnant, or had delivered within 6 weeks prior to interview. There were four emergent themes including communication, interactions with healthcare professionals, racism, and the effect of the pandemic, with further sub-themes identified. Although many of the issues identified are not unique to minority ethnic women, the findings emphasise previous study results.[20, 21, 22] The systemic inadequacies highlighted in maternity care provision for women from ethnic minority backgrounds have been exacerbated by the health service modifications resulting from the SARS-CoV-2 pandemic.

For peer review only

Strengths and limitations

This is the first qualitative study, to our knowledge, which explores the maternity experiences of women from ethnic minority backgrounds during the SARS-CoV-2 pandemic in the U.K. The interviewer, JJ, is an obstetrician, and although none of the participants were known to JJ clinically, they were aware of the interviewer's role, and it is important to acknowledge that this could have had an indirect effect on participant responses.

Interpretations

The proportion of births to migrant women has increased annually in line with growing immigration, from 11.6 percent in 1990[23] to 28.2 percent in 2019.[24] Adequate and appropriate healthcare provision for ethnic minority groups has long been recognised as integral to safe maternity care.[25] The Equality Act 2010 states that NHS treatment and care, including maternal healthcare, should be equitable and no person should be discriminated against on the basis of their ethnicity.[26] Despite recognition of issues specific to this cohort, more than a decade later, U.K. maternity care services have failed to respond to increasing levels of diversity, and as a result, ethnic minority women have continued to suffer from worse pregnancy outcomes. The Right to Health document decrees that the four key elements of universal right to healthcare are that governments must ensure that healthcare services are ethically and culturally acceptable to all, accessible to all, available in sufficient quantity, and of good quality.[27] This study provides evidence to support that development of new and innovative strategies is urgently required to guarantee that all ethnic minority women receive culturally acceptable, accessible, and equitable maternal healthcare in the U.K not only to tackle existing disparities but also to combat the additional detrimental effects of the SARS-CoV-2 pandemic.

The issues raised are not exclusive to ethnic minority women however, it is plausible that the need for support, effective communication, and good quality care are not met to a greater degree in this cohort as reported in previous studies, [28, 29] and that these long standing issues are exacerbated to a larger extent amongst these women as a result of the SARS-CoV-2 pandemic.

Good communication forms the foundation of good clinical care, and therefore, it is unsurprising that issues surrounding different aspects of communication were identified as a key theme. It is striking that although the majority of this group were fluent in English, they still identified it as a contributing problem, which mirrors previous studies' conclusions that language proficiency does not always facilitate a good pregnancy experience.[30, 31] These issues are likely to be amplified with the changes in maternal care provision during the SARS-CoV-2 pandemic, such as the predominance of telephone consultations, increasing risk of misunderstanding and misinformation. Communication, or lack thereof, played a major role in participants' perceptions of whether they were receiving acceptable

care. This consisted of routine or emergency interactions with midwives, obstetricians, general practitioners and health visitors. The majority of participants reported that regular communication with their community midwives and health visitors was excellent, however collective areas of improvement were suggested for dealings with secondary care staff during emergency visits. This requires maternity services to engage with local communities and stakeholder groups to better understand heterogeneous socio-cultural needs and to augment staff cultural competency.

Despite overlap, interactions with healthcare professionals is treated as a separate theme to communication to highlight the role of the healthcare professional in providing maternal care beyond delivery of information. A unique sub-theme that arose in this study is the effect that previous maternal care outside the U.K. has on the perception of maternal health services during the current pregnancy. Women who had not delivered elsewhere were still likely to discuss their care with friends, family, or healthcare professionals from their country of birth where practices differed. The discordance in maternal healthcare proved a source of worry, and remained unaddressed by their healthcare providers. A broader understanding of variations in maternal care is vital to provide reassurance. Although not specific to ethnic minority women, the problems of continuity of care between primary and secondary care identified in this study, are likely to be aggravated by the changes posed by the SARS-CoV-2 pandemic. Logistical problems such as inadequate or absent interpretation services or short appointment times negatively impact the relationships formed between minoritised patients and health care professionals, and represent modifiable factors influencing the holistic nature of maternity care.

As a general trend, longer duration of time spent in the U.K. was associated with awareness of racism impacting maternal health outcomes, and personal experience of racial discrimination, which is in contrast to previous findings that found that women who came to the UK more recently had a more negative perception of their care than women who had been in the UK for longer.[32] Sadly, the majority of participants were able to narrate examples of their friends, family members or wider community who had experienced racial discrimination both within and out with the context of healthcare in the U.K. Most women reported personal health behaviours that they had developed in response to others' experiences of discrimination. Previous studies have implicated patients' own experiences of racism in poor maternal health[33] however, the influence of health behaviour modification as a consequence of cognisance of discrimination that others face, as highlighted by this study, is not well explored. Previous findings show that women exposed to high levels of racism may be increased risk of adverse maternal health outcomes.[34]

All women recruited to this study had experienced a significant portion of their maternity care during lockdown restrictions due to the SARS-CoV-2 pandemic. Only two women in this study were

primiparous so the majority of participants were able to contrast their experience during this pregnancy to previous pregnancies prior to the pandemic. It is important to give due consideration to the specific challenges faced by ethnic minority women during this period such as exacerbation of communication issues and increased barriers to accessing necessary care.

Conclusion

Maternal health outcome inequalities experienced by ethnic minority groups are multifactorial in nature. With the increasingly diverse pregnant population within the U.K., tackling these discrepancies must be a priority. The first three themes reported in this study offer plausible causes to known differences in MMR of minoritised women in the U.K. compared to White women. This study provides insight into the specific challenges faced by these groups in pregnancy, which intersect with the unique problems posed by the ongoing SARS-CoV-2 pandemic to potentially widen existing ethnic disparities in maternal outcomes and experiences of maternity care. Future research should focus on in-depth exploration of maternity systems to inform the development of effective and robust interventions with the aim of reducing ethnicity based maternal health inequalities.

Keywords

Race; Ethnic minority; Pregnancy; SARS-CoV-2; Qualitative research; maternity services

Contribution to authorship

JJ was responsible for the conception of the study, planning, delivery, qualitative interviews, analysis of the study and wrote the first draft of the paper. GC and SCB contributed to the planning of the study, interpretation of results and provided critical feedback on the draft paper. All authors read and approved the final manuscript

Disclosure of interests

None declared. Completed disclosure of interests forms are available to view online as supporting information.

Details of ethics approval

The study was approved by the Research Ethics Committee of West of Scotland (20/WS/0168).

Funding

NIL to declare.

Acknowledgements

We would like to thank all of the women that participated in this study and acknowledge the help of obstetricians and midwives who assisted with recruitment.

References

1- World Health Organization; Maternal, newborn, child and adolescent health: Maternal and perinatal death reviews in the UK. Accessed 08/02/2021.

2- Knight M, Bunch K, Tuffnell D et al on behalf of MBRRACE-UK. Saving Lives, Improving Mothers' Care - Lessons learned to inform maternity care from the UK and Ireland Confidential Enquiries into Maternal Deaths and Morbidity 2016-18. Oxford: National Perinatal Epidemiology Unit, University of Oxford 2020.

3- Callaghan WM, Berg CJ. Pregnancy-related mortality among women aged 35 years and older, United States, 1991-1997. Obstetrics and Gynecology. 2003 Nov;102(5 Pt 1):1015-1021

4- Schutte, J., Steegers, E., Schuitemaker, N et al. Rise in maternal mortality in the Netherlands. BJOG: An International Journal of Obstetrics & Gynaecology, 117: 399-406.

5- Jayaweera H, Hockley CA, Redshaw ME et al. Millennium cohort study first survey.Oxford: University of Oxford; 2007

6- Marmot MG, Allen J, Goldblatt P et al. Fair society, healthy lives: strategic review of health inequalities in England post-2010.London: Institute of Health Equity; 2010

7- Lindquist, A, Kurinczuk, JJ, Redshaw et al. Experiences, utilisation and outcomes of maternity care in England among women from different socio-economic groups: findings from the 2010 National Maternity Survey. BJOG 2015; 122: 1610– 1617.

8- Lindquist A, Noor N, Sullivan E et al. The impact of socioeconomic position on severe maternal morbidity outcomes amongst women in Australia: a national case–control study. BJOG 2014; 122:1601–9.

9- Higginbottom GMA, Evans C, Morgan M et al. Interventions that improve maternity care for immigrant women in the UK: protocol for a narrative synthesis systematic review. BMJ Open. 2017 Jul 12;7(7):e016988.

10- Garcia R, Ali N, Papadopoulos C et al. Specific antenatal interventions for Black, Asian and Minority Ethnic (BAME) pregnant women at high risk of poor birth outcomes in the United Kingdom: a scoping review. BMC Pregnancy and Childbirth. 2015; 15:(1)

11- Williams DR, Mohammed SA. Discrimination and racial disparities in health: evidence and needed research. J Behav Med. 2009 Feb;32(1):20-47.

12- Henderson, J., Gao, H. & Redshaw, M. Experiencing maternity care: the care received and perceptions of women from different ethnic groups. BMC Pregnancy Childbirth 13, 196 (2013).

13- D'Souza L, Garcia J: Improving services for disadvantaged childbearing women. Child Care Health Dev. 2004, 30 (6): 599-611.

14- Knight Marian, Bunch Kathryn, Vousden Nicola et al. Characteristics and outcomes of pregnant women admitted to hospital with confirmed SARS-CoV-2 infection in UK: national population based cohort study BMJ 2020; 369 :m2107

15- Martha J Chinouya, Cathrine Madziva, Late booking amongst African women in a London borough, England: implications for health promotion, Health Promotion International, Volume 34, Issue 1, February 2019, Pages 123–132

16- Philippa L. Gardner, Penny Bunton, Dawn Edge et al. The experience of postnatal depression in West African mothers living in the United Kingdom: A qualitative study. Midwifery, Volume 30, Issue 6, 2014, pages 756-763

17- Goodwin, L, Hunter, B, Jones, A. The midwife–woman relationship in a South Wales community: Experiences of midwives and migrant Pakistani women in early pregnancy. Health Expect. 2018; 21: 347– 357

18- Jardine J, Relph S, Magee LA et al. Maternity services in the UK during the coronavirus disease 2019 pandemic: a national survey of modifications to standard care. BJOG. 2021 Apr;128(5):880-889.

19- Nowell LS, Norris JM, White DE, Moules NJ. Thematic Analysis: Striving to Meet the Trustworthiness Criteria. International Journal of Qualitative Methods. December 2017.

20- Edwards , N.P. , 2004 . Why can't women just say no? And does it really matter? In : M. Kirkham Informed choice in maternity care . Basingstoke : Palgrave MacMillan , 1 – 22 .

21- Ockleford, E.M., Berryman, J.C. and Hsu, R. 2004. Postnatal care: what new mothers say. British Journal of Midwifery, 12(3): 166–170.

22- Jomeen, J. 2010. Choice, control and contemporary childbirth: understanding through women's experiences, Oxford: Radcliffe.

23- Office for National Statistics, U.K.: Immigration, 1990. Accessed 08/02/2021

24- Office for National Statistics, U.K.: Immigration 2019. Accessed 08/02/2021

25- Deborah Turnbull, Mary McGinley, Gillian Mcllwaine, Ian A. Greer, Changing childbirth, I and II, BJOG: An International Journal of Obstetrics & Gynaecology, 10.1111/j.1471-0528.1994.tb11966.x

26- Equality Act 2010 (Commencement No.3) Order 2010.

27- World Health Organisation, Gender, equity and human rights: The right health, Factsheet No. 31, Office of the United Nations High Commissioner for Human Rights. Accessed 08/02/2021

28- Julie Jomeen & Maggie Redshaw (2013) Ethnic minority women's experience of maternity services in England, Ethnicity & Health, 18:3, 280-296

29- Maria Raisay, Jessica V. Aquino, Dawn Edge et al. Pregnancy as an ideal time for intervention to address the complex needs of black and minority ethnic women: Views of British midwives, Midwifery, Volume 31, Issue 3, 2015. Pages 373-379

30- McCourt, C. and Pearce, A. 2000. Does continuity of carer matter to women from minority ethnic groups?. Midwifery, 16: 145–154.

31- Cross-Sudworth, F., Williams, A. and Herron-Marx, S. 2011. Maternity services in multi-cultural Britain: using Q methodology to explore the views of first and second generation women of Pakistani origin. Midwifery, 27(4): 458–468.

32- Kingston D., Heaman M., Chalmers B et al. Maternity Experiences Study Group of the Canadian Perinatal Surveillance System, P.H.A.o.C. Comparison of maternity experiences of Canadian-born and recent and non-recent immigrant women: findings from the Canadian Maternity Experiences Survey. J. Obstet. Gynaecol. Can. 2011;33:1105–1115.

33- Lyons, S.M. 2008. Cultural diversity in the Dublin maternity services: the experience of maternity service providers when caring for ethnic minority women. Ethnicity and Health, 13(3): 261–276.

34- Chambers, Brittany D et al. "Exposures to structural racism and racial discrimination among pregnant and early post-partum Black women living in Oakland, California." Stress and

health : journal of the International Society for the Investigation of Stress vol. 36,2 (2020): 213-219.

	N (%)
**Ethnicity**	
Black-African	7 (43.7)
Black-Caribbean	1 (6.3)
Asian-Indian	3 (18.7)
Asian-Chinese	1 (6.3)
Asian-Bangladeshi	1 (6.3)
Asian-Pakistani	1 (6.3)
Arab	2 (12.5)
**Age**	
25 – 29	1 (6.3)
30 – 34	10 (62.5)
35 – 40	4 (24.9)
>41	1 (6.3)
**Religion**	
Christian	6 (37.5)
Muslim	7 (43.7)
Hindu	1 (6.3)
Atheist	2 (12.5)
**Country of birth**	
U.K.	1 (6.2)
Outside U.K.	15 (93.8)
**Year of immigration**	
N/A as born within U.K.	1 (6.3)
2000 - 2009	3 (18.7)
2010 - 2019	11 (68.7)
2020	1 (6.3)
**Parity**	
Nulliparous	2 (12.5)
One	6 (37.5)
Two	6 (37.5)
Three	2 (12.5)
**Antenatal/Postnatal**	
Antenatal	9 (56.3)
Postnatal	7 (43.7)
**Comorbidities**	
Yes	2 (12.5)
No	14 (87.5)
**First languages**	
Fulani	2 (12.5)
Yoruba	2 (12.5)
Arabic	1 (6.3)
Bengali	1 (6.3)
English	1 (6.3)
Farsi	1 (6.3)
French	1 (6.3)
Hindi	1 (6.3)
Igala	1 (6.3)

For peer review only

**Communication**
Respect
P1 I never feel like I am not in my country.<br>P6 I could sense that she was busy and she could have rather been off, you know, not having that one extra person popping into triage at that point. For me, it was just a case of, well, you know, I need this, so I can only apologise, but there's nowhere else I can go.<br>P11 I will also want some respect as well, because I'm, I'm capable to getting their respect. So, if other people win respect then I have to make the confidence well yes, I'm capable of getting their respect.<br>P15 I had a CVS at eleven weeks and during the procedure, I was kind of, um, emotional. Um, I was being talked to by the nurses and the midwives very well. I really appreciated the way they spoke to me.
Accent bias
P12 I don't call about everything. But when you have to call through, you're not taken seriously once your accent is heard. That's just the truth, yeah. I just feel that the…there…there's a little bit of language when it comes to…once your accent is heard, you…you…you are not the privileged lot, let me put it that way. So nobody's gonna appoint you an appointment. They don't…they don't think it's necessary
Language barrier
P8 So, therefore, if I'm given the same sort of information at the same level…with someone that is English speaking, the person that is English speaking will understand a lot better…and probably make better decisions than I would.<br>P2 It creates like a…a gap in communication where if something you express is not clearly understood so maybe they could be left with some misinterpretation<br>P9 Um, you know, when I initially came my English was not good. Eh, and it was harsh for me, the…with the way they communicated…because I don't know the language and they know; they know that I don't know the language. And I'm already depressed because I don't know whether my baby will survive or not.<br>P14 So if she feels confident to speak about the baby, to phone and speak about the issue, she, she will…yeah, she will do it. But if she feels that the person will not…she's phoning will not understand her because she won't be able to deal with the message or to say whatever she want to say, she prefers not to phone and to explore other solutions. It's really difficult sometimes…eh, to know what to do or who to speak to.
Cultural dissonance
P2 how your culture is too…can…can be a factor as well. 'Cause, umm, this can…this can cause a disparity where…a misinterpretation again where…where in my culture this is okay…But…and…and how I express myself, it might come off as you're

Jamaican Patois	1 (6.3)
Mandarin	1 (6.3)
Spanish	1 (6.3)
Telagu	1 (6.3)
Urdu	1 (6.3)
**Employment**	
Yes	13 (81.3)
No	3 (18.7)

Table 1: Study participant characteristics

For peer review only

not being accepted but, umm, it doesn't mean…it does not come off from a negativity on my side 'cause it's…it's my culture……but because someone else is from a different culture and they clash, there…there comes a…a misinterpretation, so that can affect care as well<br>P5 Um, and then the circumcision part of it is just on hold at the moment. Um, and, and that's…that's something that's quite important for us to get kind of done straightaway<br>P8 …and as a Nigerian I think people just think, a Caesarean section, no no no no,…because that has happened. So the first…the first thing I had when I have a planned C-section like, oh my gosh, have you tried? Don't you know God can heal you?<br>P11 One thing people, sometimes I find some people don't like, so well, I don't want to show people that I'm Muslim. There are lots of times because of Muslim, because of the bomb blast and other sorts of things, they are pointing me.

Table 2: Direct quotations relating to theme of communication

**Interactions with health care professionals**
Continuity of care
P2 So the continuity of care both in and outside of appointments, so it was just quite tremendous, just really good. …that has impressed me as well, and, umm, it's…it's just the thoroughness of it, to be honest, so your journey is not left at the hospital.<br>P3 And then every time a different doctor would come with a different approach……and they were not really clear and explaining to me what's going on, so I was very stressed. Um, and I really wanted to see a consultant, but I had to wait for that for quite a long time. Kind of I had to cry actually to. And I wasn't relying on the system anymore because I was hearing different opinions every time.<br>P7 So maybe they thought that I was still seen by my midwives in my, in my GP surgery or something, and they were…they never checked the size of your, um, tummy and they did not really check the position of the baby and all those kind of things.
Empathy
P3…and the midwives were very busy, um, so they couldn't attend me. And at one point, I just asked the midwife to, to look after my baby while I go to the toilet, and when I came back, she was not there and my baby had, like, vomited all over her face. So it was really, really the worst time of my life<br>P9 But the only thing what I felt was they haven't paid enough…much empathy for a human being. Eh, what I felt most was they didn't care enough to care for a human being. It was just like a product, that's what I felt. I was just like a product over there. There was no value for the emotions.<br>P12 I had a horrible…horrible experience with the…the midwives…midwives on…on duty. Okay, I've had two…I had two kids but I had no complication and so having the third one and I was, err…I…I had to go through an emergency Caesarean and all that it was very traumatic for me as a person and…but I don't think they understood that.
Informed decision making
P3 but it was mainly up to me as well to read and ask questions…rather than getting, um, like information.<br>P6 I've been given the Ready, Steady, Baby book and stuff…so there is reading materials and links and online antenatal classes, but I wouldn't say there's been that much support in terms of, you know, actually sitting me down and talking to me and explaining different things, it's been more, this is where you can go and read on stuff if you want.
Dissonance with other health care systems

For peer review only

P3 I would go and I would come back and she was shocked every time because the process in Iran is really different. I was wishing that I was actually in Iran so I could get more help from specialists.<br>P12 As much as you have to pay to get, umm, your baby delivered in America…they…they have a lot more care than in the United Kingdom.<br>P14 In Jordan she used to go every month to the doctor to check everything about the baby. She really wish if, whenever she goes to see the midwife, there is something to assure her about the baby's health…

Table 3: Direct quotations relating to theme of interaction with health care professionals

For peer review only

**Racism**
Institutional
P8 Treatment for black people will be different from treatment for white people.<br>P10 Um, they are white, they don't want to talk to you.<br>P12 Most of the time for me to get care for my children, I have to say it with a very loud voice or sounding like the child is just they're going to die in a minute before I'm responded to.
Interpersonal
P6 Um, and she said that no, this is not right, you know, um, she'd kind of gone to the GP and stuff, and she'd spoken to the midwives and they were like, oh, it's nothing that serious, but at the same time, there was white women who were having the same issue, but being taken more seriously upon.<br>P10 Yeah, because they didn't listen to us anyway. I think they listen to their people than, you know, us, and they didn't have the patience. Because I remember when I had my baby, and then I had a C-section when I was in the hospital, I was so much in pain when I called one of the midwives to come and help me to feed my baby, she was proper shouting, oh, you need to try it for yourself, you know. Just not…just not being polite. Talking to me, you know, so rude.<br>P12 in the United Kingdom they act as if you are privileged to be…so I'm…I'm also talking as, err, B…BAME, right? Like a black woman, right? They act like you're privileged that you are not having a baby in…in the bush.
Internalised
P6 so I've got a bit more I guess, a cold-headed, um, kind of, I guess, but I'm quite direct, and it's only made me, like hearing all these cases and stories and, you know, the women's experience, it only kind of makes me a bit more, I guess, hard skinned, um, just to make sure that, you know, I need to make sure that no-one is overlooking me, just for the sake of my skin.<br>P7 Well, I believe that we receive equal healthcare regardless of the colour of the skin. But what I think, the body, the nature of the body or the gene is different in compared to the people here. Our gene or the nature of our body and whatever it is quite different… Um, I, I think our bodies are much more weak or something like that maybe. Maybe less immune or less resistant, or maybe like that.<br>P11 It's not just only country, my culture, my, my view, not only religion, because we are third world country…I'm not from the developed country. When…if I'm coming from like Canada or the US or something I can, I can show that confidence

Table 4: Direct quotations relating to theme of racism

**The pandemic effect**
Isolation
P2 So COVID kind of, umm, robbed me of that experience which I'm used to back at home, where I used to attend my clinics…So clinics gave you that…that…that, umm, avenue of socialisation, meeting another mothers, new mothers……creating, umm, friendships…and…and also, you know, a continuance after birth<br>P3 I'm not getting enough support for taking care of my baby, she's crying day and night, and I couldn't have my husband or anyone because of COVID<br>P5 I had to go to all my appointments on my own.
Psychological impact
P11 COVID actually is really stressful mentally…you know. I can't go anywhere, nobody can come, which is in a pregnant woman, uh, isn't…I'd love to go somewhere, but we couldn't go anywhere because of the restriction.<br>P13 I found it very hard when you're coming to the country without knowing anyone and the coronavirus, everyone is…doing lockdown so it was very…difficult, I was very depressed. I was very anxious, yeah, umm, I feel worried a lot.
Barriers to access of care
P5 I think there were disadvantages, you don't have that face to face contact.<br>P6 I don't know if that's the pandemic causing people to be a bit more relaxed and, you know, like okay, here's the leaflet, you can go and do the reading.<br>P10 Because when I was explaining about my breathing, they didn't want to see me, they were saying that they think that I'm having a coronavirus…and I really forced, yeah, I said I'm not having a coronavirus, it's like this is… I know that this is related to my cardiomyopathy. I have really, really struggled, I can say that. I've been calling, calling, talking, talking, they don't want to see me.<br>P11 But they said, no you can't come, you have to send a picture and you have to email us. Which is sometimes, you know, is not easy to do with the phone. Because I'm not a doctor.<br>P14 she can't phone the GP to describe what is the problem. And because of the Corona, she understands that it's not easy to get an appointment. So she tried to read on the internet how she can help herself.

Table 5: Direct quotations relating to the pandemic effect

For peer review only

For peer review only

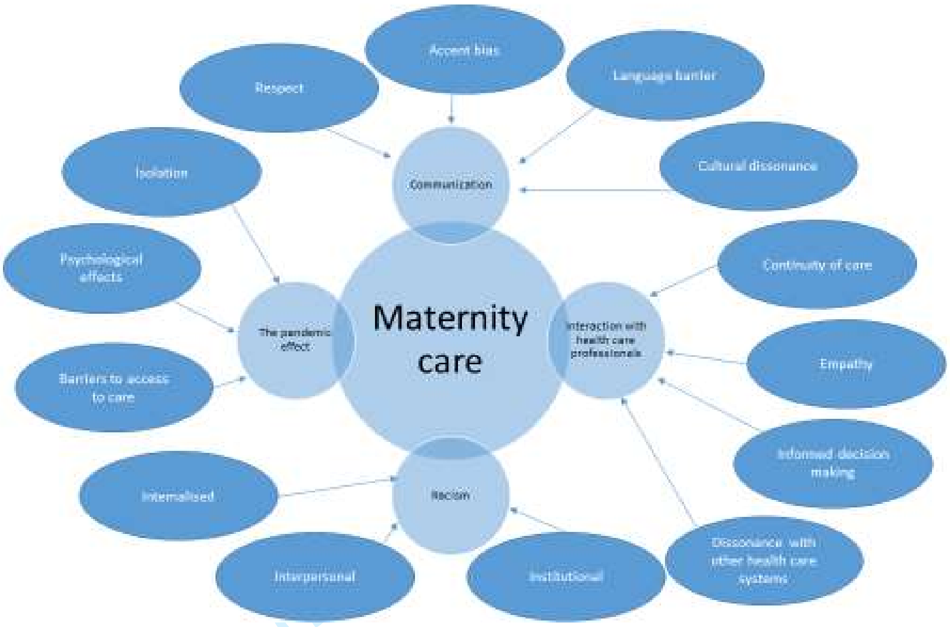

Figure 1: Themes and Subthemes

Academic and Clinical Central Office for Research and Development

PISCF 25112020 v2.0
288336

Participant Information Sheet

'A qualitative study of the lived experience of maternity care in NHS Lothian amongst Black, Asian and Minority Ethnic (BAME) women'

You are invited to take part in a research study. To help you decide whether or not to take part, it is important for you to understand why the research is being done and what it will involve. Please take time to read the following information carefully. Talk to others about the study if you wish. Contact us if there is anything that is not clear, or if you would like more information. Take time to decide whether or not you wish to take part.

What is the purpose of the study?

Pregnancy is an exciting time for many but can also be an anxious time. We are particularly interested in the experience of pregnant women from ethnic minority backgrounds and their views on the care they receive. We aim to explore people's experiences of health care, their relationships with health professionals, their experiences of their pregnancy, and their hopes and concerns for the future. We hope that this work will help change health policy for the better and improve maternity healthcare at all stages of pregnancy and birth.

Why have I been invited to take part?

You have been given this information sheet because you are currently pregnant and have identified as being from an ethnic minority group, we would like to know what your experience of healthcare has been during your pregnancy. We are conducting social research about what it is like to be a pregnant woman of ethnic minority background in NHS Lothian. This information sheet describes the research we are conducting and explains what would happen if you decided to take part in an interview.

Do I have to take part?

No, it is up to you to decide whether or not to take part. If you do decide to take part you will be given this information sheet to keep and be asked to sign a consent form. If you decide to take part you are still free to withdraw at any time without having to give a reason. Deciding not to take part or withdrawing from the study will not affect the healthcare that you receive, or your legal rights.

What will happen if I take part?

If you decide to take part, a researcher (Dr. Jeeva John, Obstetrics registrar) on the project would ask you to take part in an interview which, with your permission, will be recorded and transcribed for analysis. A pseudonym will be provided for any direct quotations that we use for publication for anonymity. The interview could take place at the hospital, online (MS Teams) or via a telephone call. Meeting face to face with social distancing and use of masks in a hospital setting would only happen if Covid-19 restrictions allow. The researcher would meet you at a time that suits you. The interview will focus on your maternity experience. We will explore your concerns, experiences,

hopes and expectations for your pregnancy especially with regards to the current Covid-19 pandemic.
We will also ask questions more generally about what you think maternity health care provision for women from minority ethnic backgrounds is like. The interview will take approximately 1 hour and you don't have to answer all the questions. We will recheck at the beginning of the interview that you are happy to continue with the interview and record your verbal consent. We would then ask you to return a completed consent form that you will receive from your midwife back to us in a prepaid envelope. If you decide to withdraw participation at any point during this process, any data or personal information you may have given us will be deleted as per your wishes. We are aiming to conduct follow up interviews later on in pregnancy to investigate how your concerns, experiences, hopes and expectations develop over time, and would ask your permission to contact you again to arrange a further interview. The timing of the follow up interview will depend on your availability and the timing of the first interview. If you do not speak English, we would arrange NHS translators to assist with interpretation during the interview.

Is there anything I need to do or avoid?

No special requirements or precautions are necessary for this type of research.

What are the possible benefits of taking part?

There are no direct benefits to you taking part in this study but the results from this study might help to improve the healthcare of pregnant womenin the future.

What are the possible disadvantages of taking part?

There are no known disadvantages if you take part in this study. Some of the topics under discussion may be emotional, and if you feel uncomfortable, you are free to stop the interview at any time. You will be advised to contact a health professional if issues of clinical concern are raised.

What if there are any problems?

If you have a concern about any aspect of this study please contact Dr. Jeeva John (07792562199, Jeeva.john@nhslothian.scot.nhs.uk) who will do her best to answer your questions.

In the unlikely event that something goes wrong and you are harmed during the research and this is due to someone's negligence then you may have grounds for a legal action for compensation against NHS Lothian but you may have to pay your legal costs. The normal National Health Service complaints mechanisms will still be available to you (if appropriate).

For peer review only - http://bmjopen.bmj.com/site/about/guidelines.xhtml

Academic and Clinical Central Office for Research and Development

PISCF 25112020 v2.0
288336

What will happen if I don't want to carry on with the study

Even if you agree to participate initially, you can change your mind at any time and withdraw from the research without having to give a reason why. You can also choose not to answer any questions without withdrawing from the study. If you decide to withdraw from the study completely and wish us to not utilise any of the information provided by yourself up until your withdrawal, all data pertaining to yourself and any interviews with you will be securely deleted.

What happens when the study is finished?

We are aiming to recruit approximately 20 women. Following each interview, the audio recordings will be contemporaneously transcribed by the lead researcher as well as a trusted transcription company (1st class secretarial services). Once the audio recordings have been transcribed, they will be immediately destroyed securely. We will store personal information including your name, contact details securely on a password protected NHS Lothian server so that we can contact you for the second interview if you consent to this, as well as give you a summary of the study findings, if you so wish. Following completion of study, this personal information will also be securely destroyed. The anonymised data following transcription will be saved for 3 years following project completion on University of Edinburgh Data SafeHaven.

Will my taking part be kept confidential?

All the information we collect during the course of the research will be kept confidential and there are strict laws which safeguard your privacy at every stage. However, if you tell us anything that may cause harm to you or someone else, we may be required to pass this information to your midwife or healthcare professional. This would only be to ensure the safety of you and other people.

How will we use information about you?

We will need to use some personal information from you for the purpose of this study.

This information will include your name, contact details, ethnicity, age, parity, relationship status and occupation. We will use this information to do the research but following completion of study, all identifiable information will be deleted, and any published material that includes transcribed data will not be identifiable to you.

We will keep all information about you safe and secure.

Once we have finished the study, we will keep some of the data so we can check the results. We will write our reports in a way that no-one can work out that you took part in the study.

For peer review only - http://bmjopen.bmj.com/site/about/guidelines.xhtml

What are your choices about how your information is used?

You can stop being part of the study at any time, without giving a reason.

Where can you find out more about how your information is used?

You can find out more about how we use your information

- at www.hra.nhs.uk/information-about-patients/
- our leaflet available from [www.hra.nhs.uk/patientdataandresearch
- by asking one of the research team
- by sending an email to Jeeva.john@nhslothian.scot.nhs.uk, or
- by ringing us on 07792562199.

What will happen to the results of the study?

This study will be written up as an article for publication in a peer reviewed journal. The results will also be available on the University of Edinburgh's Centre of Biomedicine, Self, and Society website.

You will not be identifiable from any published results.

If you wish to receive the results of the study, we will post out a summary newsletter or you can access the published summary on the Centre of Biomedicine, Self and Society website directly.

Who is organising and funding the research?

This study has been organised by the Centre of Biomedicine, Self and Society and sponsored by NHS Lothian.

The study is being funded by the Wellcome Trust.

Who has reviewed the study?

The study proposal has been reviewed by ACCORD and NHS R&D.

All research in the NHS is looked at by an independent group of people called a Research Ethics Committee. A favourable ethical opinion has been obtained from <insert REC name>. NHS Management Approval has also been given.

Researcher Contact Details

If you have any further questions about the study please contact Dr. Jeeva John on 07792562199 or email on: Jeeva.john@nhslothian.scot.nhs.uk

Independent Contact Details

If you would like to discuss this study with someone independent of the study please contact

For peer review only - http://bmjopen.bmj.com/site/about/guidelines.xhtml

accord
Academic and Clinical Central Office for Research and Development

PISCF 25112020 v2.0
288336

Martyn Pickersgill
Personal Chair of the Sociology of Science and Medicine
Martyn.pickersgill@ed.ac.uk

Complaints

If you wish to make a complaint about the study please contact:

Patient Experience Team
2 – 4 Waterloo Place, Edinburgh, EH1 3EG
feedback@nhslothian.scot.nhs.uk
0131 536 3370

For peer review only

accord
Academic and Clinical Central Office for Research and Development

NHS Lothian

PISCF 14102020 v1.0
288336

Participant ID:

CONSENT FORM

'A qualitative study of the lived experience of maternity care in NHS Lothian amongst Black, Asian and Minority Ethnic (BAME) women'

Please **initial** box

1. I confirm that I have read and understand the information sheet (14102020; v1.0) for the above study. I have had the opportunity to consider the information, ask questions and have had these questions answered satisfactorily. ☐
2. I understand that my participation is voluntary and that I am free to withdraw at any time, without giving any reason and without my medical care and/or legal rights being affected. ☐
3. I understand that relevant sections of my data collected during the study may be looked at by individuals from the Sponsor (University of Edinburgh and/or NHS Lothian), from regulatory authorities or from the NHS organisation where it is relevant to my taking part in this research. I give permission for these individuals to have access to my data. ☐
4. I give permission for my personal information (including name, address, date of birth, telephone number and consent form) to be passed to the lead researcher of the study (Dr. Jeeva John, Jeeva.john@nhslothian.scot.nhs.uk) for administration of the study. ☐
5. I understand that data collected about me during the study will be converted to anonymised data. ☐
6. I agree to my interview being audio recorded. Yes ☐ No ☐
7. I agree to my audio recorded interview being transcribed by a third party contractor. Yes ☐ No ☐
8. I agree to send this completed consent form via the provided pre-paid envelope back to the researcher. ☐
9. I agree to take part in the above study. ☐
10. I agree to being contacted later on in pregnancy to arrange a follow up interview Yes ☐ No ☐
11. I agree to receiving an end of study summary to my provided home address Yes ☐ No ☐

Name of Person Giving Consent	Date	Signature
Name of Person Receiving Consent	Date	Signature

1x original – into Site File

For peer review only - http://bmjopen.bmj.com/site/about/guidelines.xhtml

Qualitative interview topic guide

Interviews with pregnant women will be semi structured in nature but emphasis will be given to 3 main topics of:

1. Communication,
2. Access to maternity health care,
3. Cultural dissonance

Examples of questions under each sub heading will be described below:

1. Communication:
    - Are you able to communicate effectively and easily with your maternity health care providers (such as midwife/ obstetrician)?
    - Do you feel that your maternity health care providers are able to provide you with all the information and support you require in this pregnancy?
    - (Where indicated) are NHS interpreters that you have encountered so far able to answer the questions you have had relating to your care?

2. Access to maternity health care:
    - Are there any barriers to access maternity health care that you have come across especially relating to telephone triage?
    - If yes, how do you think this could be improved?
    - Have you felt that your concerns have not been listened to in this pregnancy?
    - Do you feel adequately supported if any concerns were to arise in this pregnancy?

3. Cultural dissonance:
    - Have you previously had maternity care elsewhere?
    - During this pregnancy, have you sought advice regarding health care from health care professionals outside the U.K.?
    - Do you feel that everyone in the U.K. receives equal health care regardless of their race? If not, do you have personal examples of bias in treatment or racism in health care?

Other questions that are relevant but do not fit into the themes above include:

- What are the beliefs, concerns, and expectations in relation to health of BAME women in pregnancy?
- What are their experiences of maternity care at different stages of pregnancy?
- How do women feel about being pregnant as a risk in the context of COVID-19?
- What do BAME women see as the main challenges to ensuring equality in maternity care?

For peer review only

Consolidated criteria for reporting qualitative studies (COREQ): 32-item checklist

Developed from:
Tong A, Sainsbury P, Craig J. Consolidated criteria for reporting qualitative research (COREQ): a 32-item checklist for interviews and focus groups. *International Journal for Quality in Health Care*. 2007. Volume 19, Number 6: pp. 349 – 357

YOU MUST PROVIDE A RESPONSE FOR ALL ITEMS. ENTER N/A IF NOT APPLICABLE

No. Item	Guide questions/description	Reported on Page #
**Domain 1: Research team and reflexivity**		
Personal Characteristics		
1. Inter viewer/facilitator	Which author/s conducted the interview or focus group?	3
2. Credentials	What were the researcher's credentials? E.g. PhD, MD	1
3. Occupation	What was their occupation at the time of the study?	3
4. Gender	Was the researcher male or female?	Female
5. Experience and training	What experience or training did the researcher have?	Qualitative research methodology training through University of Edinburgh
Relationship with participants		
6. Relationship established	Was a relationship established prior to study commencement?	No
7. Participant knowledge of the interviewer	What did the participants know about the researcher? e.g. personal goals, reasons for doing the research	3
8. Interviewer characteristics	What characteristics were reported about the inter viewer/facilitator? e.g. Bias, assumptions, reasons and interests in the research topic	3
**Domain 2: study design**		
Theoretical framework		
9. Methodological orientation and Theory	What methodological orientation was stated to underpin the study? e.g. grounded theory, discourse analysis, ethnography, phenomenology, content analysis	5
Participant selection		
10. Sampling	How were participants selected? e.g. purposive, convenience, consecutive,	5

For peer review only

	snowball	
11. Method of approach	How were participants approached? e.g. face-to-face, telephone, mail, email	5
12. Sample size	How many participants were in the study?	6
13. Non-participation	How many people refused to participate or dropped out? Reasons?	N/A
Setting		
14. Setting of data collection	Where was the data collected? e.g. home, clinic, workplace	5
15. Presence of non-participants	Was anyone else present besides the participants and researchers?	5
16. Description of sample	What are the important characteristics of the sample? e.g. demographic data, date	Table 1: Study participant characteristics)
Data collection		
17. Interview guide	Were questions, prompts, guides provided by the authors? Was it pilot tested?	Interview topic guide
18. Repeat interviews	Were repeat inter views carried out? If yes, how many?	No
19. Audio/visual recording	Did the research use audio or visual recording to collect the data?	Yes
20. Field notes	Were field notes made during and/or after the inter view or focus group?	During
21. Duration	What was the duration of the inter views or focus group?	30 mins – 1 hour
22. Data saturation	Was data saturation discussed?	Yes
23. Transcripts returned	Were transcripts returned to participants for comment and/or correction?	No
**Domain 3: analysis and findings**		
Data analysis		
24. Number of data coders	How many data coders coded the data?	1
25. Description of the coding tree	Did authors provide a description of the coding tree?	Yes
26. Derivation of themes	Were themes identified in advance or derived from the data?	Inductive and deductive data
27. Software	What software, if applicable, was used to manage the data?	MS Word
28. Participant checking	Did participants provide feedback on the findings?	No
Reporting		
29. Quotations presented	Were participant quotations presented to illustrate the themes/findings? Was each quotation identified? e.g. participant number	Yes
30. Data and findings consistent	Was there consistency between the data presented and the findings?	Yes
31. Clarity of major themes	Were major themes clearly presented in the findings?	Yes
32. Clarity of minor themes	Is there a description of diverse cases or	Yes

For peer review only

	discussion of minor themes?	

Once you have completed this checklist, please save a copy and upload it as part of your submission. When requested to do so as part of the upload process, please select the file type: *Checklist*. You will NOT be able to proceed with submission unless the checklist has been uploaded. Please DO NOT include this checklist as part of the main manuscript document. It must be uploaded as a separate file.

For peer review only

Exploring ethnic minority women's experiences of maternity care during the SARS-CoV-2 pandemic: a qualitative study

Journal:	*BMJ Open*
Manuscript ID	bmjopen-2021-050666.R2
Article Type:	Original research
Date Submitted by the Author:	04-Aug-2021
Complete List of Authors:	John, Jeeva; The University of Edinburgh Usher Institute of Population Health Sciences and Informatics,<br>Curry, Gwenetta; The University of Edinburgh Usher Institute of Population Health Sciences and Informatics<br>Cunningham-Burley, Sarah; The University of Edinburgh Usher Institute of Population Health Sciences and Informatics, Public Health Sciences
**Primary Subject Heading**:	Obstetrics and gynaecology
Secondary Subject Heading:	Health services research, Patient-centred medicine, Qualitative research
Keywords:	QUALITATIVE RESEARCH, COVID-19, HEALTH SERVICES ADMINISTRATION & MANAGEMENT, Organisation of health services < HEALTH SERVICES ADMINISTRATION & MANAGEMENT, Quality in health care < HEALTH SERVICES ADMINISTRATION & MANAGEMENT, OBSTETRICS

SCHOLARONE™
Manuscripts

I, the Submitting Author has the right to grant and does grant on behalf of all authors of the Work (as defined in the below author licence), an exclusive licence and/or a non-exclusive licence for contributions from authors who are: i) UK Crown employees; ii) where BMJ has agreed a CC-BY licence shall apply, and/or iii) in accordance with the terms applicable for US Federal Government officers or employees acting as part of their official duties; on a worldwide, perpetual, irrevocable, royalty-free basis to BMJ Publishing Group Ltd ("BMJ") its licensees and where the relevant Journal is co-owned by BMJ to the co-owners of the Journal, to publish the Work in this journal and any other BMJ products and to exploit all rights, as set out in our licence.

The Submitting Author accepts and understands that any supply made under these terms is made by BMJ to the Submitting Author unless you are acting as an employee on behalf of your employer or a postgraduate student of an affiliated institution which is paying any applicable article publishing charge ("APC") for Open Access articles. Where the Submitting Author wishes to make the Work available on an Open Access basis (and intends to pay the relevant APC), the terms of reuse of such Open Access shall be governed by a Creative Commons licence – details of these licences and which Creative Commons licence will apply to this Work are set out in our licence referred to above.

Other than as permitted in any relevant BMJ Author's Self Archiving Policies, I confirm this Work has not been accepted for publication elsewhere, is not being considered for publication elsewhere and does not duplicate material already published. I confirm all authors consent to publication of this Work and authorise the granting of this licence.

For peer review only

Exploring ethnic minority women's experiences of maternity care during the SARS-CoV-2 pandemic: a qualitative study

Dr. Jeeva John MBChB[1] (Corresponding author: jeeva.john@ed.ac.uk, Ph: 07792562199) Dr. Gwenetta Curry[2], Prof. Sarah Cunningham-Burley[1]

1- The University of Edinburgh Usher Institute of Population Health Sciences and Informatics, Centre for Biomedicine, Self and Society,
Edinburgh, Edinburgh, UK
2- The University of Edinburgh Usher Institute of Population Health Sciences and Informatics, Usher Institute,
Edinburgh, Edinburgh, UK

Running title: Ethnic minority maternity care during Covid-19

Abstract

Objective:

To explore the experiences of pregnancy, childbirth, antenatal and postnatal care in women belonging to ethnic minorities and to identify any specific challenges that these women faced during the SARS-CoV-2 pandemic.

Design:

This was a qualitative study using semi structured interviews of pregnant women or those who were six weeks postnatal from Black, Asian, and Minority Ethnic backgrounds. The study included sixteen women in a predominantly urban Scottish health board area.

Results

The finding are presented in four themes: 'communication', 'interactions with health care professionals', 'racism' and 'the pandemic effect'. Each theme had relevant sub-themes. 'Communication' encompassed respect, accent bias, language barrier and cultural dissonance; 'interactions with health care professionals': continuity of care, empathy, informed decision making and dissonance with other health care systems; 'racism' was deemed to be institutional, interpersonal or internalised; and 'the pandemic effect' consisted of isolation, psychological impact and barriers to access of care.

Conclusions

This study provides insight into the specific challenges faced by ethnic minority women in pregnancy, which intersect with the unique problems posed by the ongoing SARS-CoV-2 pandemic to potentially widen existing ethnic disparities in maternal outcomes and experiences of maternity care.

Funding:

This work was supported by Wellcome Trust grant number 209519/Z/17/Z.

For peer review only

Strengths and limitations of this study

- This study addresses a gap in the literature by using qualitative methods to provide an in-depth exploration of minority ethnic women's experiences of maternity care during the SARS-CoV-2 pandemic.
- This study explores the perspectives of pregnant ethnic minority women at different stages of pregnancy, as well as the postnatal period.
- A flexible topic guide was developed using existing literature, in collaboration with an obstetrician, a social scientist and a race and ethnicity lecturer, bringing together different ideologies to a complex issue.
- The interviewer is a female obstetrician, and although none of the participants were known to the interviewer clinically, they were aware of the interviewer's role, and it is important to acknowledge that this could have had an indirect effect on participant responses.

For peer review only

Introduction

Whilst the overall Maternal Mortality Rate (MMR) in the U.K. has remained relatively low over the past decade[1], the gulf in maternal outcomes between White women and women from ethnic minority backgrounds continues to expand. The latest U.K. confidential enquiry into maternal deaths (MBRRACE-U.K) report showed significant racial variations in maternal mortality. Black women were four times, while Asian women were two times as likely to die as White women during pregnancy, delivery or postpartum between 2016 and 2018.[2]

These results are comparable to the MMR in minoritised ethnic groups in the U.S.A and Netherlands despite key differences in health care systems.[3,4] Inequalities in maternal outcomes are long standing and reproducible in other high-income countries, however the causes of these disparities remain unclear. Ethnic minorities form a significant proportion of some of the most disadvantaged groups in the U.K. facing intersecting social determinants of health including unemployment and deprivation.[5,6] Although some studies have demonstrated correlation between these social elements and poor maternal outcomes, they do not fully explain the gap.[7,8]

Previous studies have investigated the experiences of pregnant immigrant women accessing maternal care in the U.K. Women who had negative experiences with health care professionals avoided accessing maternal services.[9] Unsurprisingly, not engaging with maternal care services can negatively impact both maternal and foetal health thereby increasing the risk of severe morbidity and mortality. On the contrary, women from ethnic minority backgrounds who did wish to engage, experienced limitations in accessing maternal care services in the UK, which also led to poorer health outcomes.[10] In country wide surveys of maternity care, ethnic minority women were more likely to report poor patient experience; in some cases, attributed to stereotyping and racism.[11,12] Experiences of racism and discrimination have also been linked to poor outcomes for minoritised populations.[13]

The SARS-CoV-2 pandemic has not only shone a spotlight on these disparities but may have exacerbated them.[14] A key component in establishing equality of maternal healthcare provision, is the examination of women's experiences of these services. There is little qualitative research on the lived experiences of all ethnic minority women during or immediately following pregnancy in the U.K. Previous qualitative literature in the U.K. has tended to focus on nuanced aspects of maternal care,[15] particular health conditions during pregnancy,[16] or specific ethnic groups.[17] This study aimed to explore the experiences of pregnancy, childbirth, antenatal and postnatal care in all women belonging to ethnic minority communities and to identify any specific challenges that these women faced during the SARS-CoV-2 pandemic.

For peer review only

Methods

[illegible]

Participant Characteristics and Recruitment

Women who were pregnant or within six months of delivery from a Black, Asian, and Minority ethnic background were recruited for the current study. Sixteen women were recruited: 7 Black African, 1 Black Caribbean, 3 Asian-Indian, 1 Asian-Chinese, 1 Asian-Bangladeshi, 1 Asian-Pakistani, and 2 Arab. Nine of the women were antenatal and seven were postnatal. All but one of the participants had themselves been born outside the U.K.

This study was performed in all of the community midwifery hubs and obstetric units within a predominantly urban Scottish health board. A health board is a regional authority in Scotland with local responsibility for the delivery of health services. During the SARS-CoV-2 pandemic, changes to delivery of maternal health care within this health board followed national and Royal College of Obstetricians and Gynaecologists' guidance, and included a universal shift from face-to-face consultations to telephone clinics, and restriction of visitors during hospital admission during scan appointments and following delivery.[18] Units were provided with study recruitment packs containing a participant information sheet, consent form and pre-paid envelope. Midwives were encouraged to share study information with ethnic minority patients and to provide them with recruitment packs. With their consent, participant details were securely passed onto researcher, JJ, who telephoned each patient 48 hours after receiving a recruitment pack. Participants who could not speak English were provided appropriate translations of study documents and interpreters were made available for interviews. All participants confirmed consent prior to each interview. The interview topic guide was developed by all three authors and was used to aid semi-structured interviews carried out by JJ.

Data Collection

Data collection occurred between December 2020 and January 2021. Audio-recorded interviews were transcribed verbatim by First Class Secretarial Services. Collected data was kept confidential by allocating a distinct code to each woman to protect anonymity. Data was collected until no new themes were identified and inductive and deductive thematic saturation was achieved.

Data Analysis

This study adopted a data analysis methodology based on thematic analysis.[19] The transcriptions were read and re-read, a coding frame was constructed, and the data coded to identify initial themes. A qualitative interpretive approach was taken and thematic analysis was conducted with continuous consultation between researchers JJ and GC. A consensus discussion regarding theme development was undertaken by all three authors.

For peer review only

Patient and public involvement

There was no formal PPI group that assisted with this study, and transcripts were not returned to participants for comment. However, in the course of recruitment and interviews, advice from participants and health care professionals was sought and used to create a flexible interview topic guide. A summary of study findings will be available on the Centre for Biomedicine, Self and Society for general access, and study findings will also be disseminated amongst study participants with consent having been obtained at time of interview.

Details of ethics approval

Ethical approval for this study was granted by the Research Ethics Committee of West of Scotland (20/WS/0168).

For peer review only

Results

Sixteen women participated in telephone interviews, which were up to an hour in duration (see Table 1 for sample characteristics). No follow up interviews were carried out.

Four principal themes were identified from interview data. These included communication, interactions with healthcare professionals, racism, and the pandemic effect. Each theme had subthemes, as presented in Figure 1.

Direct quotes relating to each theme are presented in Tables 2, 3, 4 and 5.

Communication

Respect

Participants who were satisfied with the maternal health system made reference to respectful communication. This was particularly important during episodes of high maternal stress (Table 2: *Respect,* P15). Participants concurred that community midwives and health visitors were considerate in their routine communication (Table 2: *Respect,* P1). A significant majority of the group conversely felt that they received disrespectful care during unsolicited hospital visits (Table 2: *Respect,* P11; P16).

Accent bias

All participants except one were first-generation immigrants with median duration of stay in the U.K. of nine years. The majority of the cohort had non "British" accents and identified bias due to accent as being a significant concern, sometimes perceived to impede access to emergency care, and prevent equality of maternal care received. One participant highlighted having a non "Westernised" accent as being interpreted as a proxy for lower socio-economic status and educational attainment, and that this was a unique barrier with regards to telephone consultations (Table 2: *Accent bias*, P12).

Language barrier

In this study, most participants spoke English fluently; only one required an interpreter. Despite the high standard of English spoken, most participants felt that language barriers were the most common cause of miscommunication between themselves and healthcare professionals. They concurrently felt they themselves were more likely to make inappropriate decisions regarding their healthcare as a result of misinterpretation (Table 2: *Language barrier*, P8). Healthcare professional misunderstanding due to language barriers was also described as a prime feature in barriers to effective communication (Table 2: *Language barrier*, P2; P9). In contrast, the participant who required an Arabic interpreter had not experienced any instances of miscommunication when an interpreter was present during pre-organised appointments. However, she admitted facing major communication challenges during unscheduled calls when an interpreter was not available. She identified a gap in recognition from healthcare professionals regarding this particular scenario, and reported that she often resorts to searching the internet for pregnancy advice even during emergencies due to anxiety surrounding communication (Table 2: *Language barrier*, P14).

Cultural dissonance

Cultural dissonance was identified as another significant barrier to effective communication. Cultural dissonance between participant and healthcare professional (Table 2: *Cultural dissonance*, P2) as well as between participants and their wider community (Table 2: *Cultural* dissonance, P8) both impacted quality of communication equally. This could be in the form of religious discordance in routine clinical practice (Table 2: *Cultural* dissonance, P11) or misunderstanding of wider cultural context (Table 2: *Cultural dissonance*, P5).

Interactions with healthcare professionals

Quality of interaction with healthcare professionals was generally associated with four key domains: continuity of care; empathy; informed decision making; and dissonance with previous experiences of maternity care. This theme, although strongly interlinked with the previous one, was derived separately to emphasise the non-verbal, and institutional aspects of health care interactions that affected participants' experiences of maternity care.

For peer review only

Continuity of care

Overall, participants felt they received good continuity of care throughout their pregnancy. Primiparous women particularly valued routine postnatal check-ups (Table 3: *Continuity of care*, P2). A common sentiment that arose in women requiring regular input from secondary care during the antenatal period was ineffective communication between their community midwives and hospital midwives or obstetricians, and vice-versa, sometimes resulting in omission of crucial clinical information (Table 3: *Continuity of care*, P7). In the women who experienced prolonged hospital admissions, the inability to discuss ongoing care with the same healthcare professionals led to varying information being given to the patient leading to dissolution of trust (Table 3: *Continuity of care*, P3).

Empathy

The most important aspect of interactions with healthcare professionals was the presence of empathy. In instances where the healthcare professional was deemed indifferent, participants often cited being hurried, feeling unheard, and uncared for, which negatively impacted the interaction, and the overall perception of maternity care (Table 3: *Empathy*, P3). Participants who experienced apathy in previous pregnancies recognised that the possibility of recurrence was a source of anxiety throughout subsequent pregnancies (Table 3: *Empathy*, P9; P12).

Informed decision making

Although all participants reported some level of information provision from healthcare providers regarding clinical decision making, almost everyone agreed that they would benefit from more thorough discussions. Most participants received information about their pregnancy in the form of signposting to books or websites but they expressed that their individual information needs would have been better met by one-to-one discussions (Table 3: *Informed decision making*: P6). Women who felt that their questions remained unanswered did not feel involved in shared decision making (Table 3: *Informed decision making:* P3).

Dissonance with other healthcare systems

Fifty percent of the study population had experienced maternity care outside the present health board, with a significant proportion having delivered outside the U.K. All participants who previously delivered outside the U.K. had experienced privatised healthcare.

Access to early pregnancy care, and shared decision making were some of the reasons provided by women who previously delivered in other parts of the U.K. reporting higher satisfaction with the study health board in the current pregnancy. The majority of the participants who had delivered abroad felt that their previous pregnancy experiences were generally better compared to care received

For peer review only

in this health board. Reasons given for this discordance varied amongst individual participants, and included; improved access to ultrasound scans, availability of medical specialists, and treatment by healthcare providers (Table 3: *Dissonance with other healthcare systems*, P12; P14). A high proportion of primiparous women had sought advice from relatives, friends and/or healthcare professionals in their country of birth (Table 3: *Dissonance with other healthcare systems*, P3).

Racism

Institutional

Institutional racism was highlighted as a significant issue in pregnancy care by most of the participants. A small proportion of Black participants concomitantly worked in healthcare, and were able to provide examples of racial discrimination against themselves from their colleagues (Table 4: *Institutional*, P10). Some of the participants expressed distrust in maternal healthcare due to their concerns that medical research and treatments are tailored for their White counterparts (Table 4: *Institutional*, P8). Participants who had experienced institutional racism were also likely to perceive barriers to accessing healthcare compared to White women (Table 4: *Institutional*, P12).

Interpersonal

Most women gave examples of acts of racism against their friends, or family rather than themselves (Table 4: *Interpersonal*, P6; P12).

Internalised

In participants who expressed internalised racist beliefs, these almost always affected health behaviours. In one example, a significant delay in diagnosis of a common condition led one participant to believe that the main problem was her lack of assertiveness compared to White women (Table 4: *Internalised*, P6). In another case, a participant felt that she did not have the right to question the healthcare she was receiving, being originally from a low-income country (Table 4: *Internalised,* P11). Yet another participant strongly believed that disparities in maternal health outcomes could be explained by physiological differences between ethnic groups (Table 4: *Internalised*, P7).

The pandemic effect

This study was developed in order to better understand the lived experiences of women from ethnic minority backgrounds during maternity care. Due to the time period that the study was conducted in, the SARS-CoV-2 pandemic and its' direct and indirect consequences were naturally brought up by all participants. The sub-themes below illustrate the most common impacts of the pandemic as mentioned by the participants.

For peer review only

Isolation

Most women reported feeling isolated during their pregnancy due to features specific to the SARS-CoV-2 pandemic. This was particularly a problem for those who felt that they would have benefitted from the presence of a companion when important information relating to their pregnancy was being relayed to them (Table 5: *Isolation*, P5). Although most women were understanding of the limitations posed by the pandemic, a significant proportion expressed loneliness exacerbated by not being able to engage with their usual pregnancy support networks, and did not feel that virtual groups mitigated this effect (Table 5: *Isolation,* P2; P3).

Psychological impact

The detrimental effect of the pandemic on mental health during pregnancy was highlighted by all participants. Women expressed feeling more anxious, fearful, and lacking autonomy in comparison to their previous pregnancies. This sentiment was particularly emphasized by the participants who had little or no family members nearby (Table 5: *Psychological impact*, P11; P13). The majority of participants reported that they were not routinely asked about their mental health in relation to the pandemic by healthcare professionals. Fewer still were signposted to appropriate support groups for help.

Barriers to access of care

Most participants had some of their routine face-to-face appointments replaced by telephone calls. The majority felt that virtual appointments were not as effective, especially during unscheduled care (Table 5: *Barriers to access of care*, P5). Women expressed uncertainty surrounding the accuracy of information relayed via telephone. (Table 5: *Barriers to access of care*, P6). In circumstances where participants did seek physical consultations, they experienced barriers, and often had to repeatedly call in order to be seen (Table 5: *Barriers to access of care*, P10; P11). On the contrary, some women delayed seeking medical help due to apprehensions surrounding contracting SARS-CoV-2 (Table 5: *Barriers to access of care*, P14).

Discussion

Main findings

This study focussed on the lived experiences during the SARS-CoV-2 pandemic of minority ethnic women who were pregnant, or had delivered within 6 weeks prior to interview. There were four

For peer review only

emergent themes including communication, interactions with healthcare professionals, racism, and the effect of the pandemic, with further sub-themes identified. Although many of the issues identified are not unique to minority ethnic women, the findings emphasise previous study results.[20, 21, 22] The systemic inadequacies highlighted in maternity care provision for women from ethnic minority backgrounds have been exacerbated by the health service modifications resulting from the SARS-CoV-2 pandemic.

Strengths and limitations

This is the first qualitative study, to our knowledge, which explores the maternity experiences of women from ethnic minority backgrounds during the SARS-CoV-2 pandemic in the U.K.

This study is methodologically limited by reliance on narratives, which are often subject to recall bias, from a small number of patients at a single Scottish health board, and may therefore restrict generalisability of results. However, we believe that our inclusive selection criteria provided a degree of homogeneity in participant experiences, which was helpful in facilitating rich discussion. Moreover, delivery within the same health board provided a foundation from which varying experiences could be compared and contrasted.

In addition, we acknowledge that although ethnic differences are important study factors, they are inevitably intertwined with socioeconomic factors. Ethnic minority groups with a higher socio-economic position are possibly over represented in this study. Selection bias cannot be excluded as a higher socio-economic status is associated with increased utilisation of maternity care services, which is how participant recruitment took place. Thus, views expressed by participants in this study are likely to be under representative of the true challenges in accessing maternal health care faced by minority ethnic pregnant women from lower socio-economic backgrounds in other parts of the U.K.

The interviewer, JJ, is an obstetrician, and although none of the participants were known to JJ clinically, they were aware of the interviewer's role, and it is important to acknowledge that this could have had an indirect effect on participant responses. Finally, the research team's different positionalities in clinical and public health backgrounds may have influenced data interpretation. However, we believe that this diversity broadened and deepened our understanding of these women's experiences.

Interpretations

The proportion of births to migrant women has increased annually in line with growing immigration, from 11.6 percent in 1990[23] to 28.2 percent in 2019.[24] Adequate and appropriate healthcare provision

for ethnic minority groups has long been recognised as integral to safe maternity care.[25] The Equality Act 2010 states that NHS treatment and care, including maternal healthcare, should be equitable and no person should be discriminated against on the basis of their ethnicity.[26] Despite recognition of issues specific to this cohort, more than a decade later, U.K. maternity care services have failed to respond to increasing levels of diversity, and as a result, ethnic minority women have continued to suffer from worse pregnancy outcomes. The Right to Health document decrees that the four key elements of universal right to healthcare are that governments must ensure that healthcare services are ethically and culturally acceptable to all, accessible to all, available in sufficient quantity, and of good quality.[27] This study provides evidence to support that development of new and innovative strategies is urgently required to guarantee that all ethnic minority women receive culturally acceptable, accessible, and equitable maternal healthcare in the U.K not only to tackle existing disparities but also to combat the additional detrimental effects of the SARS-CoV-2 pandemic.

The issues raised are not exclusive to ethnic minority women however, it is plausible that the need for support, effective communication, and good quality care are not met to a greater degree in this cohort as reported in previous studies, [28, 29] and that these long standing issues are exacerbated to a larger extent amongst these women as a result of the SARS-CoV-2 pandemic.

Good communication forms the foundation of good clinical care, and therefore, it is unsurprising that issues surrounding different aspects of communication were identified as a key theme. It is striking that although the majority of this group were fluent in English, they still identified it as a contributing problem, which mirrors previous studies' conclusions that language proficiency does not always facilitate a good pregnancy experience.[30, 31] These issues are likely to be amplified with the changes in maternal care provision during the SARS-CoV-2 pandemic, such as the predominance of telephone consultations, increasing risk of misunderstanding and misinformation. Communication, or lack thereof, played a major role in participants' perceptions of whether they were receiving acceptable care. This consisted of routine or emergency interactions with midwives, obstetricians, general practitioners and health visitors. The majority of participants reported that regular communication with their community midwives and health visitors was excellent, however collective areas of improvement were suggested for dealings with secondary care staff during emergency visits. This requires maternity services to engage with local communities and stakeholder groups to better understand heterogeneous socio-cultural needs and to augment staff cultural competency.

Despite overlap, interactions with healthcare professionals is treated as a separate theme to communication to highlight the role of the healthcare professional in providing maternal care beyond delivery of information. A unique sub-theme that arose in this study is the effect that previous maternal care outside the U.K. has on the perception of maternal health services during the current

pregnancy. Women who had not delivered elsewhere were still likely to discuss their care with friends, family, or healthcare professionals from their country of birth where practices differed. The discordance in maternal healthcare proved a source of worry, and remained unaddressed by their healthcare providers. A broader understanding of variations in maternal care is vital to provide reassurance. Although not specific to ethnic minority women, the problems of continuity of care between primary and secondary care identified in this study, are likely to be aggravated by the changes posed by the SARS-CoV-2 pandemic. Logistical problems such as inadequate or absent interpretation services or short appointment times negatively impact the relationships formed between minoritised patients and health care professionals, and represent modifiable factors influencing the holistic nature of maternity care.

As a general trend, longer duration of time spent in the U.K. was associated with awareness of racism impacting maternal health outcomes, and personal experience of racial discrimination, which is in contrast to previous findings that found that women who came to the UK more recently had a more negative perception of their care than women who had been in the UK for longer.[32] Sadly, the majority of participants were able to narrate examples of their friends, family members or wider community who had experienced racial discrimination both within and out with the context of healthcare in the U.K. Most women reported personal health behaviours that they had developed in response to others' experiences of discrimination. Previous studies have implicated patients' own experiences of racism in poor maternal health[33] however, the influence of health behaviour modification as a consequence of cognisance of discrimination that others face, as highlighted by this study, is not well explored. Previous findings show that women exposed to high levels of racism may be increased risk of adverse maternal health outcomes.[34]

All women recruited to this study had experienced a significant portion of their maternity care during lockdown restrictions due to the SARS-CoV-2 pandemic. Only two women in this study were primiparous so the majority of participants were able to contrast their experience during this pregnancy to previous pregnancies prior to the pandemic. It is important to give due consideration to the specific challenges faced by ethnic minority women during this period such as exacerbation of communication issues and increased barriers to accessing necessary care.

Conclusion

Maternal health outcome inequalities experienced by ethnic minority groups are multifactorial in nature. With the increasingly diverse pregnant population within the U.K., tackling these discrepancies must be a priority. The first three themes reported in this study offer plausible causes to

known differences in MMR of minoritised women in the U.K. compared to White women. This study provides insight into the specific challenges faced by these groups in pregnancy, which intersect with the unique problems posed by the ongoing SARS-CoV-2 pandemic to potentially widen existing ethnic disparities in maternal outcomes and experiences of maternity care. Future research should focus on in-depth exploration of maternity systems to inform the development of effective and robust interventions with the aim of reducing ethnicity based maternal health inequalities.

Keywords

Race; Ethnic minority; Pregnancy; SARS-CoV-2; Qualitative research; maternity services

Contribution to authorship

JJ was responsible for the conception of the study, planning, delivery, qualitative interviews, analysis of the study and wrote the first draft of the paper. GC and SCB contributed to the planning of the study, interpretation of results and provided critical feedback on the draft paper. All authors read and approved the final manuscript

Data availability statement

All data relevant to the study are included in the article or uploaded as supplementary information.

Disclosure of interests

None declared. Completed disclosure of interests forms are available to view online as supporting information.

Details of ethics approval

The study was approved by the Research Ethics Committee of West of Scotland (20/WS/0168).

Funding

This work was supported by Wellcome Trust grant number 209519/Z/17/Z.

Acknowledgements

We would like to thank all of the women that participated in this study and acknowledge the help of obstetricians and midwives who assisted with recruitment.

Figure 1: Themes and Subthemes

References

1- World Health Organization; Maternal, newborn, child and adolescent health: Maternal and perinatal death reviews in the UK. Accessed 08/02/2021.

2- Knight M, Bunch K, Tuffnell D et al on behalf of MBRRACE-UK. Saving Lives, Improving Mothers' Care - Lessons learned to inform maternity care from the UK and Ireland Confidential Enquiries into Maternal Deaths and Morbidity 2016-18. Oxford: National Perinatal Epidemiology Unit, University of Oxford 2020.

3- Callaghan WM, Berg CJ. Pregnancy-related mortality among women aged 35 years and older, United States, 1991-1997. Obstetrics and Gynecology. 2003 Nov;102(5 Pt 1):1015-1021

4- Schutte, J., Steegers, E., Schuitemaker, N et al. Rise in maternal mortality in the Netherlands. BJOG: An International Journal of Obstetrics & Gynaecology, 117: 399-406.

5- Jayaweera H, Hockley CA, Redshaw ME et al. Millennium cohort study first survey.Oxford: University of Oxford; 2007

6- Marmot MG, Allen J, Goldblatt P et al. Fair society, healthy lives: strategic review of health inequalities in England post-2010.London: Institute of Health Equity; 2010

7- Lindquist, A, Kurinczuk, JJ, Redshaw et al. Experiences, utilisation and outcomes of maternity care in England among women from different socio-economic groups: findings from the 2010 National Maternity Survey. BJOG 2015; 122: 1610– 1617.

8- Lindquist A, Noor N, Sullivan E et al. The impact of socioeconomic position on severe maternal morbidity outcomes amongst women in Australia: a national case–control study. BJOG 2014; 122:1601–9.

9- Higginbottom GMA, Evans C, Morgan M et al. Interventions that improve maternity care for immigrant women in the UK: protocol for a narrative synthesis systematic review. BMJ Open. 2017 Jul 12;7(7):e016988.

10- Garcia R, Ali N, Papadopoulos C et al. Specific antenatal interventions for Black, Asian and Minority Ethnic (BAME) pregnant women at high risk of poor birth outcomes in the United Kingdom: a scoping review. BMC Pregnancy and Childbirth. 2015; 15:(1)

11- Williams DR, Mohammed SA. Discrimination and racial disparities in health: evidence and needed research. J Behav Med. 2009 Feb;32(1):20-47.

12- Henderson, J., Gao, H. & Redshaw, M. Experiencing maternity care: the care received and perceptions of women from different ethnic groups. BMC Pregnancy Childbirth 13, 196 (2013).

13- D'Souza L, Garcia J: Improving services for disadvantaged childbearing women. Child Care Health Dev. 2004, 30 (6): 599-611.

14- Knight Marian, Bunch Kathryn, Vousden Nicola et al. Characteristics and outcomes of pregnant women admitted to hospital with confirmed SARS-CoV-2 infection in UK: national population based cohort study BMJ 2020; 369 :m2107

15- Martha J Chinouya, Cathrine Madziva, Late booking amongst African women in a London borough, England: implications for health promotion, Health Promotion International, Volume 34, Issue 1, February 2019, Pages 123–132

16- Philippa L. Gardner, Penny Bunton, Dawn Edge et al. The experience of postnatal depression in West African mothers living in the United Kingdom: A qualitative study. Midwifery, Volume 30, Issue 6, 2014, pages 756-763

17- Goodwin, L, Hunter, B, Jones, A. The midwife–woman relationship in a South Wales community: Experiences of midwives and migrant Pakistani women in early pregnancy. Health Expect. 2018; 21: 347– 357

18- Jardine J, Relph S, Magee LA et al. Maternity services in the UK during the coronavirus disease 2019 pandemic: a national survey of modifications to standard care. BJOG. 2021 Apr;128(5):880-889.

19- Nowell LS, Norris JM, White DE, Moules NJ. Thematic Analysis: Striving to Meet the Trustworthiness Criteria. International Journal of Qualitative Methods. December 2017.

20- Edwards , N.P. , 2004 . Why can't women just say no? And does it really matter? In : M. Kirkham Informed choice in maternity care . Basingstoke : Palgrave MacMillan , 1 – 22 .

21- Ockleford, E.M., Berryman, J.C. and Hsu, R. 2004. Postnatal care: what new mothers say. British Journal of Midwifery, 12(3): 166–170.

22- Jomeen, J. 2010. Choice, control and contemporary childbirth: understanding through women's experiences, Oxford: Radcliffe.

23- Office for National Statistics, U.K.: Immigration, 1990. Accessed 08/02/2021

24- Office for National Statistics, U.K.: Immigration 2019. Accessed 08/02/2021

25- Deborah Turnbull, Mary McGinley, Gillian Mcllwaine, Ian A. Greer, Changing childbirth, I and II, BJOG: An International Journal of Obstetrics & Gynaecology, 10.1111/j.1471-0528.1994.tb11966.x

26- Equality Act 2010 (Commencement No.3) Order 2010.

27- World Health Organisation, Gender, equity and human rights: The right health, Factsheet No. 31, Office of the United Nations High Commissioner for Human Rights. Accessed 08/02/2021

28- Julie Jomeen & Maggie Redshaw (2013) Ethnic minority women's experience of maternity services in England, Ethnicity & Health, 18:3, 280-296

29- Maria Raisay, Jessica V. Aquino, Dawn Edge et al. Pregnancy as an ideal time for intervention to address the complex needs of black and minority ethnic women: Views of British midwives, Midwifery, Volume 31, Issue 3, 2015. Pages 373-379

30- McCourt, C. and Pearce, A. 2000. Does continuity of carer matter to women from minority ethnic groups?. Midwifery, 16: 145–154.

31- Cross-Sudworth, F., Williams, A. and Herron-Marx, S. 2011. Maternity services in multi-cultural Britain: using Q methodology to explore the views of first and second generation women of Pakistani origin. Midwifery, 27(4): 458–468.

32- Kingston D., Heaman M., Chalmers B et al. Maternity Experiences Study Group of the Canadian Perinatal Surveillance System, P.H.A.o.C. Comparison of maternity experiences of Canadian-born and recent and non-recent immigrant women: findings from the Canadian Maternity Experiences Survey. J. Obstet. Gynaecol. Can. 2011;33:1105–1115.

33- Lyons, S.M. 2008. Cultural diversity in the Dublin maternity services: the experience of maternity service providers when caring for ethnic minority women. Ethnicity and Health, 13(3): 261–276.

34- Chambers, Brittany D et al. "Exposures to structural racism and racial discrimination among pregnant and early post-partum Black women living in Oakland, California." Stress and

health : journal of the International Society for the Investigation of Stress vol. 36,2 (2020): 213-219.

	N (%)
**Ethnicity**	
Black-African	7 (43.7)
Black-Caribbean	1 (6.3)
Asian-Indian	3 (18.7)
Asian-Chinese	1 (6.3)
Asian-Bangladeshi	1 (6.3)
Asian-Pakistani	1 (6.3)
Arab	2 (12.5)
**Age**	
25 – 29	1 (6.3)
30 – 34	10 (62.5)
35 – 40	4 (24.9)
>41	1 (6.3)
**Religion**	
Christian	6 (37.5)
Muslim	7 (43.7)
Hindu	1 (6.3)
Atheist	2 (12.5)
**Country of birth**	
U.K.	1 (6.2)
Outside U.K.	15 (93.8)
**Year of immigration**	
N/A as born within U.K.	1 (6.3)
2000 - 2009	3 (18.7)
2010 - 2019	11 (68.7)
2020	1 (6.3)
**Parity**	
Nulliparous	2 (12.5)
One	6 (37.5)
Two	6 (37.5)
Three	2 (12.5)
**Antenatal/Postnatal**	
Antenatal	9 (56.3)
Postnatal	7 (43.7)
**Comorbidities**	
Yes	2 (12.5)
No	14 (87.5)
**First languages**	
Fulani	2 (12.5)
Yoruba	2 (12.5)
Arabic	1 (6.3)
Bengali	1 (6.3)
English	1 (6.3)
Farsi	1 (6.3)
French	1 (6.3)
Hindi	1 (6.3)
Igala	1 (6.3)

For peer review only

**Communication**
Respect
P1 I never feel like I am not in my country.<br>P6 I could sense that she was busy and she could have rather been off, you know, not having that one extra person popping into triage at that point. For me, it was just a case of, well, you know, I need this, so I can only apologise, but there's nowhere else I can go.<br>P11 I will also want some respect as well, because I'm, I'm capable to getting their respect. So, if other people win respect then I have to make the confidence well yes, I'm capable of getting their respect.<br>P15 I had a CVS at eleven weeks and during the procedure, I was kind of, um, emotional. Um, I was being talked to by the nurses and the midwives very well. I really appreciated the way they spoke to me.
Accent bias
P12 I don't call about everything. But when you have to call through, you're not taken seriously once your accent is heard. That's just the truth, yeah. I just feel that the…there…there's a little bit of language when it comes to…once your accent is heard, you…you…you are not the privileged lot, let me put it that way. So nobody's gonna appoint you an appointment. They don't…they don't think it's necessary
Language barrier
P8 So, therefore, if I'm given the same sort of information at the same level…with someone that is English speaking, the person that is English speaking will understand a lot better…and probably make better decisions than I would.<br>P2 It creates like a…a gap in communication where if something you express is not clearly understood so maybe they could be left with some misinterpretation<br>P9 Um, you know, when I initially came my English was not good. Eh, and it was harsh for me, the…with the way they communicated…because I don't know the language and they know; they know that I don't know the language. And I'm already depressed because I don't know whether my baby will survive or not.<br>P14 So if she feels confident to speak about the baby, to phone and speak about the issue, she, she will…yeah, she will do it. But if she feels that the person will not…she's phoning will not understand her because she won't be able to deal with the message or to say whatever she want to say, she prefers not to phone and to explore other solutions. It's really difficult sometimes…eh, to know what to do or who to speak to.
Cultural dissonance
P2 how your culture is too…can…can be a factor as well. 'Cause, umm, this can…this can cause a disparity where…a misinterpretation again where…where in my culture this is okay…But…and…and how I express myself, it might come off as you're

Jamaican Patois	1 (6.3)
Mandarin	1 (6.3)
Spanish	1 (6.3)
Telagu	1 (6.3)
Urdu	1 (6.3)
**Employment**	
Yes	13 (81.3)
No	3 (18.7)

Table 1: Study participant characteristics

For peer review only

not being accepted but, umm, it doesn't mean…it does not come off from a negativity on my side 'cause it's…it's my culture……but because someone else is from a different culture and they clash, there…there comes a…a misinterpretation, so that can affect care as well<br>P5 Um, and then the circumcision part of it is just on hold at the moment. Um, and, and that's…that's something that's quite important for us to get kind of done straightaway<br>P8 …and as a Nigerian I think people just think, a Caesarean section, no no no no,…because that has happened. So the first…the first thing I had when I have a planned C-section like, oh my gosh, have you tried? Don't you know God can heal you?<br>P11 One thing people, sometimes I find some people don't like, so well, I don't want to show people that I'm Muslim. There are lots of times because of Muslim, because of the bomb blast and other sorts of things, they are pointing me.

Table 2: Direct quotations relating to theme of communication

For peer review only

**Interactions with health care professionals**
Continuity of care
P2 So the continuity of care both in and outside of appointments, so it was just quite tremendous, just really good. …that has impressed me as well, and, umm, it's…it's just the thoroughness of it, to be honest, so your journey is not left at the hospital.<br>P3 And then every time a different doctor would come with a different approach……and they were not really clear and explaining to me what's going on, so I was very stressed. Um, and I really wanted to see a consultant, but I had to wait for that for quite a long time. Kind of I had to cry actually to. And I wasn't relying on the system anymore because I was hearing different opinions every time.<br>P7 So maybe they thought that I was still seen by my midwives in my, in my GP surgery or something, and they were…they never checked the size of your, um, tummy and they did not really check the position of the baby and all those kind of things.
Empathy
P3…and the midwives were very busy, um, so they couldn't attend me. And at one point, I just asked the midwife to, to look after my baby while I go to the toilet, and when I came back, she was not there and my baby had, like, vomited all over her face. So it was really, really the worst time of my life<br>P9 But the only thing what I felt was they haven't paid enough…much empathy for a human being. Eh, what I felt most was they didn't care enough to care for a human being. It was just like a product, that's what I felt. I was just like a product over there. There was no value for the emotions.<br>P12 I had a horrible…horrible experience with the…the midwives…midwives on…on duty. Okay, I've had two…I had two kids but I had no complication and so having the third one and I was, err…I…I had to go through an emergency Caesarean and all that it was very traumatic for me as a person and…but I don't think they understood that.
Informed decision making
P3 but it was mainly up to me as well to read and ask questions…rather than getting, um, like information.<br>P6 I've been given the Ready, Steady, Baby book and stuff…so there is reading materials and links and online antenatal classes, but I wouldn't say there's been that much support in terms of, you know, actually sitting me down and talking to me and explaining different things, it's been more, this is where you can go and read on stuff if you want.
Dissonance with other health care systems

P3 I would go and I would come back and she was shocked every time because the process in Iran is really different. I was wishing that I was actually in Iran so I could get more help from specialists.<br>P12 As much as you have to pay to get, umm, your baby delivered in America…they…they have a lot more care than in the United Kingdom.<br>P14 In Jordan she used to go every month to the doctor to check everything about the baby. She really wish if, whenever she goes to see the midwife, there is something to assure her about the baby's health…

Table 3: Direct quotations relating to theme of interaction with health care professionals

**Racism**
Institutional
P8 Treatment for black people will be different from treatment for white people.<br>P10 Um, they are white, they don't want to talk to you.<br>P12 Most of the time for me to get care for my children, I have to say it with a very loud voice or sounding like the child is just they're going to die in a minute before I'm responded to.
Interpersonal
P6 Um, and she said that no, this is not right, you know, um, she'd kind of gone to the GP and stuff, and she'd spoken to the midwives and they were like, oh, it's nothing that serious, but at the same time, there was white women who were having the same issue, but being taken more seriously upon.<br>P10 Yeah, because they didn't listen to us anyway. I think they listen to their people than, you know, us, and they didn't have the patience. Because I remember when I had my baby, and then I had a C-section when I was in the hospital, I was so much in pain when I called one of the midwives to come and help me to feed my baby, she was proper shouting, oh, you need to try it for yourself, you know. Just not…just not being polite. Talking to me, you know, so rude.<br>P12 in the United Kingdom they act as if you are privileged to be…so I'm…I'm also talking as, err, B…BAME, right? Like a black woman, right? They act like you're privileged that you are not having a baby in…in the bush.
Internalised
P6 so I've got a bit more I guess, a cold-headed, um, kind of, I guess, but I'm quite direct, and it's only made me, like hearing all these cases and stories and, you know, the women's experience, it only kind of makes me a bit more, I guess, hard skinned, um, just to make sure that, you know, I need to make sure that no-one is overlooking me, just for the sake of my skin.<br>P7 Well, I believe that we receive equal healthcare regardless of the colour of the skin. But what I think, the body, the nature of the body or the gene is different in compared to the people here. Our gene or the nature of our body and whatever it is quite different… Um, I, I think our bodies are much more weak or something like that maybe. Maybe less immune or less resistant, or maybe like that.<br>P11 It's not just only country, my culture, my, my view, not only religion, because we are third world country…I'm not from the developed country. When…if I'm coming from like Canada or the US or something I can, I can show that confidence

Table 4: Direct quotations relating to theme of racism

For peer review only

**The pandemic effect**
Isolation
P2 So COVID kind of, umm, robbed me of that experience which I'm used to back at home, where I used to attend my clinics…So clinics gave you that…that…that, umm, avenue of socialisation, meeting another mothers, new mothers……creating, umm, friendships…and…and also, you know, a continuance after birth<br>P3 I'm not getting enough support for taking care of my baby, she's crying day and night, and I couldn't have my husband or anyone because of COVID<br>P5 I had to go to all my appointments on my own.
Psychological impact
P11 COVID actually is really stressful mentally…you know. I can't go anywhere, nobody can come, which is in a pregnant woman, uh, isn't…I'd love to go somewhere, but we couldn't go anywhere because of the restriction.<br>P13 I found it very hard when you're coming to the country without knowing anyone and the coronavirus, everyone is…doing lockdown so it was very…difficult, I was very depressed. I was very anxious, yeah, umm, I feel worried a lot.
Barriers to access of care
P5 I think there were disadvantages, you don't have that face to face contact.<br>P6 I don't know if that's the pandemic causing people to be a bit more relaxed and, you know, like okay, here's the leaflet, you can go and do the reading.<br>P10 Because when I was explaining about my breathing, they didn't want to see me, they were saying that they think that I'm having a coronavirus…and I really forced, yeah, I said I'm not having a coronavirus, it's like this is… I know that this is related to my cardiomyopathy. I have really, really struggled, I can say that. I've been calling, calling, talking, talking, they don't want to see me.<br>P11 But they said, no you can't come, you have to send a picture and you have to email us. Which is sometimes, you know, is not easy to do with the phone. Because I'm not a doctor.<br>P14 she can't phone the GP to describe what is the problem. And because of the Corona, she understands that it's not easy to get an appointment. So she tried to read on the internet how she can help herself.

Table 5: Direct quotations relating to the pandemic effect

For peer review only

For peer review only

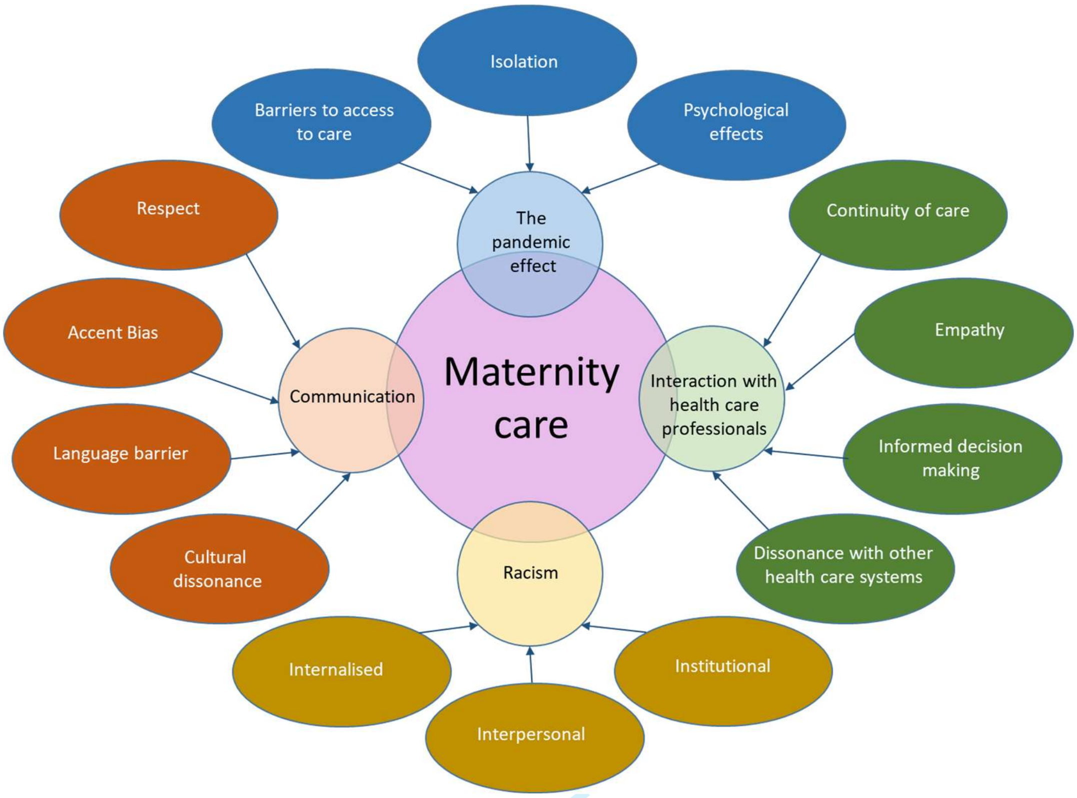

Figure 1: Themes and Subthemes

Consolidated criteria for reporting qualitative studies (COREQ): 32-item checklist

Developed from:
Tong A, Sainsbury P, Craig J. Consolidated criteria for reporting qualitative research (COREQ): a 32-item checklist for interviews and focus groups. International Journal for Quality in Health Care. 2007. Volume 19, Number 6: pp. 349 – 357

YOU MUST PROVIDE A RESPONSE FOR ALL ITEMS. ENTER N/A IF NOT APPLICABLE

No. Item	Guide questions/description	Reported on Page #
Domain 1: Research team and reflexivity		
Personal Characteristics		
1. Inter viewer/facilitator	Which author/s conducted the interview or focus group?	5
2. Credentials	What were the researcher's credentials? E.g. PhD, MD	1
3. Occupation	What was their occupation at the time of the study?	3
4. Gender	Was the researcher male or female?	3
5. Experience and training	What experience or training did the researcher have?	3
Relationship with participants		
6. Relationship established	Was a relationship established prior to study commencement?	3
7. Participant knowledge of the interviewer	What did the participants know about the researcher? e.g. personal goals, reasons for doing the research	3
8. Interviewer characteristics	What characteristics were reported about the inter viewer/facilitator? e.g. Bias, assumptions, reasons and interests in the research topic	3
Domain 2: study design		
Theoretical framework		
9. Methodological orientation and Theory	What methodological orientation was stated to underpin the study? e.g. grounded theory, discourse analysis, ethnography, phenomenology, content analysis	5
Participant selection		

For peer review only

10. Sampling	How were participants selected? e.g. purposive, convenience, consecutive, snowball	5
11. Method of approach	How were participants approached? e.g. face-to-face, telephone, mail, email	5
12. Sample size	How many participants were in the study?	6
13. Non-participation	How many people refused to participate or dropped out? Reasons?	N/A
Setting		
14. Setting of data collection	Where was the data collected? e.g. home, clinic, workplace	5
15. Presence of nonparticipants	Was anyone else present besides the participants and researchers?	5
16. Description of sample	What are the important characteristics of the sample? e.g. demographic data, date	19
Data collection		
17. Interview guide	Were questions, prompts, guides provided by the authors? Was it pilot tested?	5
18. Repeat interviews	Were repeat inter views carried out? If yes, how many?	6
19. Audio/visual recording	Did the research use audio or visual recording to collect the data?	5
20. Field notes	Were field notes made during and/or after the inter view or focus group?	5
21. Duration	What was the duration of the inter views or focus group?	6
22. Data saturation	Was data saturation discussed?	5
23. Transcripts returned	Were transcripts returned to participants for comment and/or correction?	6
Domain 3: analysis and findings		
Data analysis		
24. Number of data coders	How many data coders coded the data?	5
25. Description of the coding tree	Did authors provide a description of the coding tree?	5
26. Derivation of themes	Were themes identified in advance or derived from the data?	5
27. Software	What software, if applicable, was used to manage the data?	5
28. Participant checking	Did participants provide feedback on the findings?	6
Reporting		

For peer review only

29. Quotations presented	Were participant quotations presented to illustrate the themes/findings? Was each quotation identified? e.g. participant number	6, 21, 22, 23, 24
30. Data and findings consistent	Was there consistency between the data presented and the findings?	N/A
31. Clarity of major themes	Were major themes clearly presented in the findings?	6
32. Clarity of minor themes	Is there a description of diverse cases or	6
	discussion of minor themes?	

Once you have completed this checklist, please save a copy and upload it as part of your submission. When requested to do so as part of the upload process, please select the file type: Checklist. You will NOT be able to proceed with submission unless the checklist has been uploaded. Please DO NOT include this checklist as part of the main manuscript document. It must be uploaded as a separate file.

For peer review only
